# *Jeongeuplla avenae* gen. nov., sp. nov., a novel β-carotene-producing bacterium that alleviates salinity stress in Arabidopsis

**DOI:** 10.3389/fmicb.2023.1265308

**Published:** 2023-12-06

**Authors:** Lingmin Jiang, Yuxin Peng, Ki-Hyun Kim, Doeun Jeon, Hanna Choe, Ah-Reum Han, Cha Young Kim, Jiyoung Lee

**Affiliations:** ^1^Biological Resource Center, Korean Collection for Type Cultures (KCTC), Korea Research Institute of Bioscience and Biotechnology (KRIBB), Jeongeup, Republic of Korea; ^2^Advanced Radiation Technology Institute, Korea Atomic Energy Research Institute, Jeongeup, Republic of Korea; ^3^Department of Biosystems and Bioengineering, KRIBB School of Biotechnology, University of Science and Technology (UST), Daejeon, Republic of Korea

**Keywords:** polyphasic taxonomy, salt stress, Arabidopsis, β-carotene, reactive oxygen species

## Abstract

A novel endophytic bacterium, designated DY-R2A-6^T^, was isolated from oat (*Avena sativa* L.) seeds and found to produces β-carotene. Phylogenetic analysis based on 16S rRNA gene sequences revealed that strain DY-R2A-6^T^ had 96.3% similarity with *Jiella aquimaris* LZB041^T^, 96.0% similarity with *Aurantimonas aggregate* R14M6^T^ and *Aureimonas frigidaquae* JCM 14755^T^, and less than 95.8% similarity with other genera in the family *Aurantimonadaceae*. The complete genome of strain DY-R2A-6^T^ comprised 5,929,370 base pairs, consisting of one full chromosome (5,909,198 bp) and one plasmid (20,172 bp), with a G + C content was 69.1%. The overall genome-related index (OGRI), including digital DNA–DNA hybridization (<20.5%), ANI (<79.2%), and AAI (<64.2%) values, all fell below the thresholds set for novel genera. The major cellular fatty acids (>10%) of strain DY-R2A-6^T^ were C_16:0_, C_19:0_ cyclo ω8c, and summed feature 8 (C_18:1_ω7c and/or C_18:1_ω6c). Ubiquinone-10 was the main respiratory quinone. We identified the gene cluster responsible for carotenoid biosynthesis in the genome and found that the pink-pigment produced by strain DY-R2A-6^T^ is β-carotene. In experiment with Arabidopsis seedlings, co-cultivation with strain DY-R2A-6^T^ led to a 1.4-fold increase in plant biomass and chlorophyll content under salt stress conditions, demonstrating its capacity to enhance salt stress tolerance in plants. Moreover, external application of β-carotene to Arabidopsis seedlings under salt stress conditions also mitigated the stress significantly. Based on these findings, strain DY-R2A-6^T^ is proposed to represent a novel genus and species in the family *Aurantimonadaceae*, named *Jeongeuplla avenae* gen. nov., sp. nov. The type strain is DY-R2A-6^T^ (= KCTC 82985^T^ = GDMCC 1.3014^T^). This study not only identified a new taxon but also utilized genome analysis to predict and confirm the production of β-carotene by strain DY-R2A-6^T^. It also demonstrated the ability of this strain to enhance salt stress tolerance in plants, suggesting potential application in agriculture to mitigate environmental stress in crops.

## 1 Introduction

Reactive oxygen species (ROS), including singlet oxygen (^1^O_2_), superoxide (O_2_^−^), hydroxyl radicals (OH), and hydrogen peroxide (H_2_O_2_), are well-established agents that can cause oxidative damage to cellular structure (Apel and Hirt, 2004). Nevertheless, in the face of abiotic and biotic stress response, organisms must effectively utilize ROS as signaling molecules in various biological processes. Consequently, all living organisms must carefully regulate the delicate balance between ROS production and ROS quenching to maintain optimal cellular function. Increased sodium (Na^+^) levels in plant cells disrupt the balance between ROS production and scavenging, leading to ROS accumulation (Miller et al., 2010). This accumulation subsequently causes oxidative stress and cellular damage (Ling et al., 2009; Miller et al., 2010). Simultaneously, ROS plays a pivotal role as important secondary messenger in root tissues, precisely controlling cytoplasmic proliferation and differentiation, which has a significant impact on root growth and development (He et al., 2012; Jiang et al., 2012).

To protect cells from harmful compounds, bacteria have evolved an enzyme known as superoxide dismutase (SOD) to continuously neutralize endogenously generated ROS by converting O_2_^−^ into molecular oxygen (O_2_) and hydrogen peroxide (H_2_O_2_), and removing H_2_O_2_ through catalase and peroxidase enzymes (Imlay, 2006). Bacteria also have evolved the ability to produce carotenoids as a means of protection against UV radiation and oxidative damage, which allows them to survive in such diverse environments as seawater and bark. There are over 1,200 different carotenoids found in various sources, with 722 source organisms reported in the carotenoid database.^1^ Bacteria can produce 324 carotenoids. The best-known carotenoid-producing bacteria include *Flavobacterium* spp., *Agrobacterium* spp., and *Pseudomonas* spp., with carotenoids such as canthaxanthin, zeaxanthin, astaxanthin, β-carotene, and lycopene having application in food, feed, cosmetics, and disease prevention (Ram et al., 2020; Lopez et al., 2023). Recent studies have focused on carotenoid biosynthesis via fermentation by microbes such as microalgae (*Dunaliella salina*), fungi (*Blakeslea trispora*), and yeast (*Xanthophyllomyces dendrorhous*) (Verdoes et al., 2003; Pourkarimi et al., 2020; Papadaki and Mantzouridou, 2021; Lopez et al., 2023).

The family *Aurantimonadaceae* belongs to the order *Rhizobiales* and was established and named *Aurantimonadaceae* fam. nov. in 2020 after the delimitation of the *Rhizobiaceae* and *Mycoplana* taxa from *Aurantimonadaceae* (Kuykendall, 2005). The family comprises the genera *Aurantimonas* (the type genus), *Consotaella*, *Fulvimarina*, *Jiella*, and *Mangrovicella* (Hördt et al., 2020).^2^ Bacteria within the family *Aurantimonadaceae* are typically Gram-stain-negative, rod-shaped, and mesophilic, and have varying NaCl requirements. They can be either motile via flagella or sessile and are catalase- and oxidase-positive. Ubiquinone is the major quinone, the main constituent fatty acids are C_18:1_ ω7c and C_19:0_ cyclo ω8c, and the most abundant polar lipids are phosphatidylglycerol, diphosphatidylglycerol, phosphatidylcholine, phosphatidylethanolamine, and phosphatidylmonomethylethanolamine (Hördt et al., 2020). Members of this family have been found in a wide range of environments, including seawater (Liang et al., 2015), plant philosopher and endophytes (Li et al., 2018; Chen et al., 2022), hydrothermal plumes (Ren et al., 2014), soil (Lin et al., 2013), lichens (Zhang K. et al., 2020), ice cores (Guo et al., 2017), and coral (Denner et al., 2003). Carotenoids are often present in members of this family, which produce pigments of varying brownish-yellow shades (Cho and Giovannoni, 2003), yellow (Li et al., 2017), pale yellow (Denner et al., 2003), and golden orange (Denner et al., 2003).

Although some species in family *Aurantimonadaceae* are known to produce colored pigments, only one study to date has described the carotenoids produced by this family, which have spectral absorption peaks at 477, 453, and 419 nm (Cho and Giovannoni, 2003). The carotenoid biosynthetic apparatus in this family, including the biosynthetic gene clusters (BGCs), has not been extensively studied, and the associated genes remain to be comprehensively characterized. With the advancements in sequencing technologies, putative BGCs, which encode secondary metabolites such as carotenoids, can be identified via whole-genome sequencing analysis using tools such as antiSMASH (Blin et al., 2021). BGCs include non-ribosomal peptide synthase (NRPS), type I and type II polyketide synthase (PKS), lanthipeptides, lasso peptides, sactipeptides, and thiopeptides. β-carotene, a carotenoid, is a red-orange pigment belonging to the terpenoid family with the molecular formula C_40_H_56_. The beneficial properties of β-carotene include antioxidant and anticancer effects and the prevention of night blindness, and it is also widely used as a food colorant (Dufossé et al., 2005; Lai et al., 2014; Virtamo et al., 2014). In plants, meanwhile, β-carotene has multiple known functions, including defending against free radical-induced cell damage and mitigating salt stress (Zhang et al., 2012, 2016; Ren et al., 2021; Song et al., 2022).

Oat (*Avena sativa* L.) is an important cereal grain despite accounting for only 1% of global cereal production. Oats are used both as animal feed and human food owing to their nutritional value and added health benefits. Oats are a rich source of essential nutrients, such as proteins, dietary fiber, various B vitamins, multiple dietary minerals, and β-glucans (Joyce et al., 2019; Lee et al., 2021). Research has shown that oats can help alleviate diabetes symptoms (Tapola et al., 2005), enhance insulin sensitivity (Bao et al., 2014), and prevent cancer (Li et al., 2022). Therefore, it is important to conduct research to expand the scope of oat cultivation. Recent research has shown that plant growth-promoting rhizobacteria play a pivotal role in enhancing salt tolerance in oats (Zhang et al., 2022). In this study, we isolated a total of 37 endophytic strains from surface-sterilized oat seeds and, following further polyphasic taxonomic analysis, identified a novel bacterial strain, DY-R2A-6^T^. This strain was found to belong to a new genus in the family *Aurantimonadaceae*. Genome mining via antiSMASH led to the detection of a gene cluster for carotenoid biosynthesis in DY-R2A-6^T^ genome, and a pink-yellow color pigment produced by this bacterium was identified as β-carotene. We found that this β-carotene-producing strain, DY-R2A-6^T^, can alleviate salt stress in Arabidopsis by mitigating oxidative damage and scavenging reactive oxygen species (ROS).

## 2 Materials and methods

### 2.1 Sample collection and isolation

Strain DY-R2A-6^T^ was isolated from oat cultivar “Dae Yang” (*Avena sativa* L.) grown in Jeongeup, Jeollabuk-do, Republic of Korea (35°30′29.8″N, 126°50′13.9″E). The seeds were surface-sterilized with 1% sodium hypochlorite and ground in 1× PBS. Serial dilutions were prepared, spread on R2A agar (Difco), and incubated at 25°C for 7 days. Colonies based on phenotypic and color differences were picked and subsequently streaked on fresh medium. Among the 37 endophytes obtained, strain DY-R2A-6^T^, a circular bacterium exhibiting a red-orange pigment, was selected for further study. The strain was deposited at the Korean Collection for Type Cultures (KCTC) under accession number KCTC 82985^T^ and at the Guandong Microbial Culture Collection Center (GDMCC) under accession number GDMCC 1.3014^T^. The strain was preserved in 10% (w/v) sterile skimmed milk at −80°C and grown on R2A medium for 4 days for subsequent tests unless otherwise stated.

### 2.2 Physiological and biochemical characterization

Detailed cell shapes and flagella were examined both by scanning electron microscopy (FE-SEM, Regulus 8100; Hitachi) and transmission electron microscopy (TEM, H-7600; Hitachi). To determine cellular motility, R2A medium containing 0.4% agar was used. The Gram reaction was performed using a Gram staining kit (Difco). Catalase activity was determined by observing bubble production after the addition of a 3% (v/v) H_2_O_2_ solution to fresh cells and oxidase activity was determined using an oxidase reagent kit (bioMérieux; Korea). Strain DY-R2A-6^T^ was cultured on different media, including R2A, NA, PDA, TSA, LB, and MA, for 7 days at 25°C to optimize growth conditions. The optimal temperature was determined by growing the strain at temperatures ranging from 4 to 60°C (4, 10, 15, 20, 25, 30, 37, 40, 45, 50, 55, and 60°C). NaCl tolerance was determined by growing the strain in R2A liquid medium containing 0 to 15% (w/v; 1% increments) concentrations of NaCl for 7 days at 25°C with shaking and then measuring the optical density at 600 nm using a microplate spectrophotometer (Multiskan SkyHigh, Thermo Fisher Scientific). Meanwhile, the pH tolerance was tested by growing the strain on a R2A solution of pH 3 to 12, adjusted with 1N HCl or NaOH, and subsequently recording the optical density at 600 nm. Biochemical characteristics, including substrate utilization, acid production, carbohydrate contents, and enzyme activities, were tested using API 20NE, API ZYM (NaCl 0.85% medium), and API 50CH (bioMérieux; Korea) according to the manufacturer’s protocols. All closely related type strains were tested under the same conditions.

The chemotaxonomic properties of strain DY-R2A-6^T^ examined included cellular fatty acids, polar lipids, and quinones. To analyze the cellular fatty acid composition, cells of strain DY-R2A-6^T^ in the exponential growth phase were harvested and subjected to saponification, methylation, and extraction according to the standard MIDI Sherlock Microbial Identification System (version 6.0). Gas chromatography–mass spectrometry (Model 6890N; Agilent) was employed to analyze the prepared fatty acids using the Microbial Identification software package (Sasser, 2006) and the Sherlock Aerobic Bacterial Database (TSBA 6.1).

Isoprenoid quinones were extracted from 100 mg of freeze-dried cells using a chloroform/methanol mixture (2:1, v/v) and quantified using reverse-phase high-performance liquid chromatography with detection of UV absorbance at 275 nm. Meanwhile, polar lipids were extracted from 100 mg of freeze-dried cells using a chloroform/methanol mixture (1:2, v/v) and separated by two-dimensional thin-layer chromatography on Kieselgel 60F254 plates (silica gel, 10 × 10 cm; Merck). The TLC plates were developed first with a solvent comprising chloroform: methanol: DW (65:25:4, v/v) and then with a second solvent consisting of chloroform: methanol: acetic acid: water (80, 12:15:4, v/v). For the detection of lipids, 0.2% ninhydrin (Sigma-Aldrich), α-naphthol, molybdenum blue (Sigma-Aldrich), 4% phosphomolybdic acid reagent, and Dragendorff’s solution were used to detect amino group-containing lipids, sugar-containing lipids, phosphorus-containing lipids, total lipids, and quarternary nitrogen-containing lipids, respectively.

### 2.3 16S rRNA gene sequence analysis

Sequencing of the 16S rRNA gene of strain DY-R2A-6^T^ was performed by Macrogen, Inc.^3^ Bacterial DNA was used as the template and the universal primers 27F and 1492R were used for PCR amplification. The PCR product was then sequenced using the 27F, 1492R, and 800R primers (Senthilraj et al., 2016). The 16S rRNA gene sequence data were assembled using Vector NTI software (1.6.1) yielding the near-full-length sequence of the 16S rRNA gene. The closest neighbors of the 16S rRNA gene sequence were identified by BLAST-based comparison against all strains with a validated name in the GenBank^4^ and the EzBioCloud databases^5^ (Yoon et al., 2017). The 16S rRNA gene sequences of all validated members in the family *Aurantimonadaceae* obtained from LPSN and GenBank were multiply aligned using the BioEdit program and a phylogenetic tree was constructed using Molecular Evolutionary Genetics Analysis (MEGA 11.0) software (Tamura et al., 2021) employing NJ, ME, and ML algorithms with 1,000 bootstrap iterations. The *Rhodospirillum rubrum* DSM 467^T^ was used as an outgroup.

### 2.4 Nucleic acid extraction and whole-genome sequencing, assembly, and annotation

Genomic DNA was extracted as previously described (Jiang et al., 2022). After purification, the DNA was sequenced using the PacBio sequel system and the Illumina sequencing platform at the Macrogen facility (Macrogen, Korea), and assembled *de novo* using the Microbial Assembly application (v8.0). The assembled genome was assessed for quality, completeness, and contamination using the MiGA web server (Rodriguez et al., 2018). Genome annotation was undertaken using the Rast SEED, PGAP of NCBI (Tatusova et al., 2016). The OGRI (dDDH, ANI, and AAI values) between strain DY-R2A-6^T^ and closely-related strains was determined using a variety of tools, including the Genome-to-Genome Distance Calculation (GGDC) webserver^6^ (Meier-Kolthoff et al., 2013), the AAI calculator^7^ (Konstantinidis and Tiedje, 2005), the ANI calculator^8^, and the standalone OAT software (Lee et al., 2016). A graphical circular map of the DY-R2A-6^T^ genome was created using CGView.^9^ To identify potential secondary metabolites, metabolic pathways were reconstructed using BlastKOALA, a website based on the Kyoto Encyclopedia of Genes and Genomes (KEGG) pathway database (Kanehisa et al., 2016). The presence of secondary metabolite BGCs was analyzed using the antiSMASH tool^10^ (Blin et al., 2021). Finally, to determine the phylogenomic relatedness of strain DY-R2A-6^T^ within the family *Aurantimonadacea*e, a whole-genome phylogenetic analysis (based on 92 core genes) was conducted using the UBCG pipeline, as described by Na et al. (2018). The *Rhodospirillum rubrum* DSM 467^T^ was used as an outgroup. The TYGS was also used for taxonomic classification^11^ (Meier-Kolthoff and Goker, 2019).

### 2.5 Carotenoid structure determination

When grown on R2A medium, strain DY-R2A-6^T^ exhibited carotenoid accumulation, resulting in the production of a pink-yellow pigment. To extract the total carotenoids, fresh cells (100 mg) were mixed with 1 mL of methanol (HPLC grade) and sonicated for 10 min. The methanol extract was then centrifuged at 8,000 rpm for 10 min and the supernatant was collected and passed through a 0.2-μm membrane filter. The UV–Vis absorption spectrum (200–800 nm) of the total carotenoid extracts was recorded using a microplate spectrophotometer (Multiskan SkyHigh, Thermo Fisher Scientific). The obtained data were compared with those of standards and published values.

To further identify the pigment, the methanol extract was analyzed using an HPLC system (Shimadzu Corporation, Kyoto, Japan) equipped with a YMC carotenoid column (250 × 4.6 mm.D, S-5 μm, YMC, Japan). The separation column was maintained at a temperature of 35°C, with solvent A, consisting of methanol: MTBE: water (85, 10:5, v/v), and solvent B, consisting of 100% MTBE, as the mobile phase. The flow rate was set to 1 mL/min, the wavelength was set at 450 nm, and a 20-μL sample was injected and analyzed at EZmass, Inc. (Korea).^12^

### 2.6 Plant growth conditions

Seeds from the *Arabidopsis thaliana* transgenic line DR5::GUS were sterilized with 1% sodium hypochlorite for 15 min, rinsed three times with sterile distilled water for 5 min, and stored at 4°C for 1 day. The sterilized seeds were then planted on ½ MS medium (2.2 g/L MS powder, 10 g/L sucrose, 0.5 g/L2-MES, 15 g/L bacto-agar) and the plants were grown in a growth chamber at 22°C with 55–60% relative humidity under a 16-h light/8-h dark cycle to facilitate germination. Seven-day-old seedlings were co-cultured with strain DY-R2A-6^T^ or control (distilled water) for 10 days on 1/2 MS medium with or without NaCl (100 mM). To assess plant growth conditions, leaf fresh weight, root fresh weight, and chlorophyll content were measured (formula: *y* = 8.02 × A_663_ + 20.2 × A_645_). Statistical analysis was performed to compare differences between untreated controls and co-cultivation with the strain DY-R2A-6^T^ under normal (0 mM NaCl) or saline conditions (100 mM NaCl). The GraphPad Prism program (version 8.2.1) was used to perform the Sidak’s multiple comparison tests followed by two-way analysis of variance (ANOVA). In addition, a β-carotene solutions (50 μM) were evenly sprayed on the seedlings and allowed to incubate for an additional 10 days. Three different replicate sets were performed for all treatments, each consisting of six plants. The results presented in this study represent the mean values obtaining from 18 plants and their respective standard deviations (±SD).

### 2.7 ROS detection

ROS in plant roots were detected using DCFH-DA (Sigma, Korea) as previously described (Jiang et al., 2023), but with a minor modification. Briefly, the seedlings were exposed to 5 μM DCFH-DA in 50 mM sodium phosphate buffer (pH 7.0) for 30 min in the dark. Subsequently, the roots were rinsed three times with water and imaged under a fluorescence microscope (Zeiss Imager.A2) equipped with a FITC filter as described by Jiang et al. (2023). The means of the sum intensities of fluorescence in the roots were determined using ImageJ software (National Institute of Health, MD, USA).

### 2.8 Statistical analysis

All experiments were conducted two or three times independently. Multiple comparisons using two-way ANOVA were performed with GraphPad Prism (version 8.2.1).

### 2.9 Data availability

Strain DY-R2A-6^T^ can be obtained from the Korean Collection for Type Cultures (KCTC 82985^T^) and the Guangdong Microbial Culture Collection Center (GDMCC 1.3014^T^). The sequence of the 16S rRNA gene of strain DY-R2A-6^T^ can be found under GenBank accession number OL709414.1. The accession numbers for the whole-genome sequence of strain DY-R2A-6^T^ are CP113520.1–CP113521.1. The associated BioSample and BioProject accession numbers are SAMN31843394 and PRJNA678113, respectively. The taxonomy ID for strain DY-R2A-6^T^ is 2998449.

## 3 Results and discussion

### 3.1 Characterization of phenotypic and biochemical traits

Strain DY-R2A-6^T^ was isolated from surface-sterilized oat seeds and grew well on Reasoner’s 2A (R2A) medium, but not potato dextrose agar (PDA), Luria-Bertani agar (LB), marine agar 2216 (MA), nutrient agar (NA), or trypticase soy agar (TSA) medium. Further characterization revealed that strain DY-R2A-6^T^ was Gram-negative, weak-motile, had a short-rod or ovoid shape (0.9–1.1 × 0.9–2.2 μm, Supplementary Figure S1), and lophotrichous. The colonies formed by the strain were creamy pink to yellowish and ranged in diameter from 1 to 4 mm. Regarding the optimal growth conditions, strain DY-R2A-6^T^ grew best at a temperature of 20–25°C, but could grow at temperatures ranging from 10 to 30°C. The optimal pH for growth was 7 but ranged from pH 6–10. DY-R2A-6^T^ could tolerate NaCl concentrations of up to 2.5% NaCl, with optimal growth seen at 1.5%. Strain DY-R2A-6^T^ was catalase- and oxidase-positive, which is a common characteristic of Gram-negative bacteria. Additional characteristics that distinguished strain DY-R2A-6^T^ from other closely-related strains are shown in Table 1.

**Table 1:** Differential physiological and biochemical characteristics of strain DY-R2A-6^T^ relative to phylogenetically related strains.

Characteristic	1	2	3	4
Isolation source	Oat seeds	Ross sea^a^	Water-cooling system^b^	Surface seawater^c^
Colony color	Pink to yellowish	Pale yellow^a^	Yellow^b^	Yellow^c^
Motility	Weak	Non-gliding^a^	Motile^b^	Motile^c^
Cell size (μm)	0.9–1.1 × 0.9–2.2	0.7–1 × 1.1–1.5^a^	0.6–0.8 × 0.8–1.2^b^	0.6–0.7 × 2.3–2.8^c^
Growth conditions				
Temperature range, °C; (optimum range)	10–30 (20–25)	4–36 (25–30)^a^	15–37 (25)^b^	4–42 (28–37)^c^
NaCl tolerance, % (*w*/*v*); (optimum %)	0–2.5 (1.5)	1–15 (3)^a^	0–7 (0)^b^	0–15 (1–7)^c^
pH range (optimum)	6–10 (7)	6.0–8.3 (7)^a^	5–11 (7)^b^	7–10 (7–8)^c^
Optimum medium	R2A	MA^a^	TSA, NA, and R2A^b^	MA and R2A^c^
Oxidase test	Aerobic	Aerobic^a^	Facultative anaerobic^b^	Aerobic^c^
Colony size (mm)	1–4	1^a^	3^b^	0.5–1^c^
DNA G + C content (mol %)	69.1	67.4^a^	63.9^b^	71.3^c^
API 20NE				
Glucose assimilation	+	−	+	−
Arabinose assimilation	+	−	−	−
Mannose assimilation	−	−	+	−
Mannotol assimilation	−	−	+	−
Gluconate glusomine	−	−	+	−
Malate glusomine	−	−	+	−
Acid production (API 50CH)	−	−	−	−
Erythritol	−	−	+	−
D-Arabinose	−	−	+	−
L-Arabinose	+	−	+	−
D-Ribose	+	−	+	−
D-Xylose	+	−	+	−
D-Adonitol	+	−	+	−
Methyl-βD-xylopyranoside	+	−	+	−
D-Galactose	+	−	+	−
D-Glucose	+	−	+	−
D-Fructose	+	−	+	−
D-Mannose	+	−	+	−
L-Sorbose	−	−	+	−
L-Rhamnose	+	−	−	−
Inositol	−	−	+	−
D-Mannitol	+	+	+	−
D-Fucose	+	−	+	−
L-Fucose	−	−	+	−
D-Arabitol	−	−	+	−
L-Arabitol	−	−	+	−
API ZYM				
Valine arylamidase	+	−	w	−
Cystine arylamidase	+	+	w	−
Trypsin	+	−	+	+
α-glucosidase	−	−	−	+

Strains: (1) *Jeongeuplla avenae* DY-R2A-6^T^; (2) *Aurantimonas aggregata* KCTC 52919^T^; (3) *Aureimonas frigidaquae* KCTC 12893^T^; and (4) *Jiella aquimaris* JCM 30119^T^. All data are from the present study unless otherwise indicated. +, positive; w, weakly positive; −, negative.

^a^Li et al. (2017); ^b^Kim et al. (2008); ^c^Liang et al. (2015).

### 3.2 Molecular characterization studies

**Figure 1:**
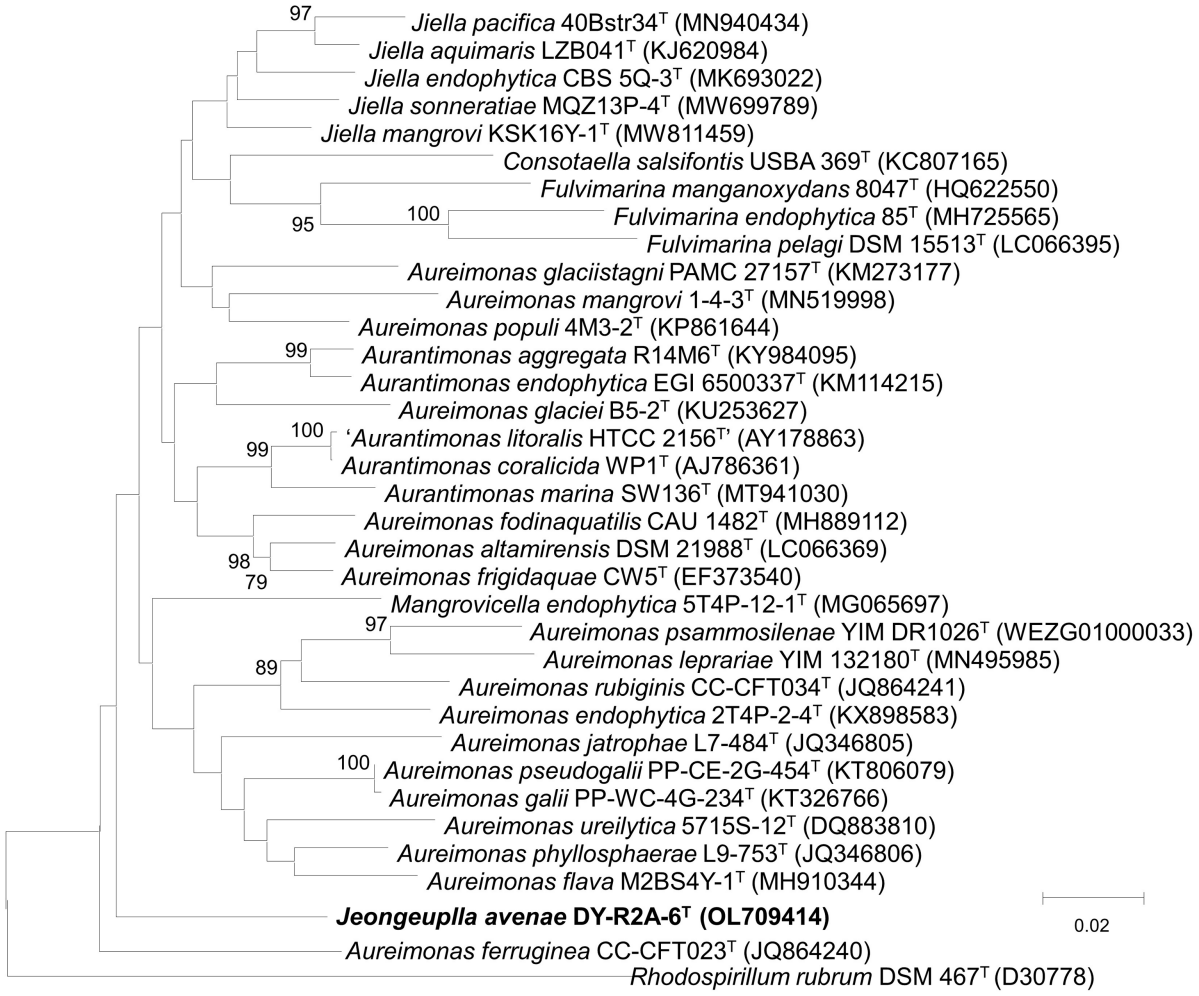
Neighbor-joining algorithm-based phylogenetic tree based on the 16S rRNA gene of strain DY-R2A-6^T^. The numbers displayed at the nodes represent bootstrap values of 70% and above. *Rhodosirillum rubrum* DSM 467^T^ was used as outgroup. The bar indicates a scale of 0.02 nucleotide substitutions per nucleotide position.

The 16S rRNA gene sequence was obtained through Sanger sequencing as well as extraction from the whole genome. The sequence was identified using the GenBank (see text footnote 4) and the EzBioCloud databases (see text footnote 4) (Yoon et al., 2017). The near-full-length 16S rRNA amplicon of strain DY-R2A-6^T^ was 1,401 nucleotides long (OL709414). Based on initial identification, genera *Jiella*, *Aurantimonas*, and *Aureimonas* in the family *Aurantimonadaceae* were identified as the closest related species, but with 16S rRNA sequences similarity ≤96.3%. The closest phylogenetic neighbors for strain DY-R2A-6^T^ were *Jiella aquimaris* LZB041^T^ (96.3%), *Aurantimonas aggregata* R14M6^T^ (96.0%), and *Aureimonas frigidaquae* JCM 14755^T^ (96.0%). To clarify the taxonomic position of strain DY-R2A-6^T^, all the genera of the family *Aurantimonadaceae* were included in the phylogenetic analysis based on the 16S rRNA gene sequence. The resulting neighbor-joining (NJ) tree indicated that strain DY-R2A-6^T^ formed a distinct cluster within this family and stood apart from any genera within the family (Figure 1). This result was supported by maximum likelihood (ML) and minimum evolution (ME) algorithm-based phylogenetic trees. Based on a novel species recognition threshold of 98.6% (Kim M. et al., 2014), and given that the three 16S rRNA genes with the greatest similarities belonged to different genera, strain DY-R2A-6^T^ could not be assigned to any validly published taxon at either the species or genus level. Phylogenetic analysis clearly showed that strain DY-R2A-6^T^ belonged to the family *Aurantimonadaceae*, but its phylogenetic position was distant from that of other related genera within the family.

### 3.3 Phylogenomic analyses

**Figure 2:**
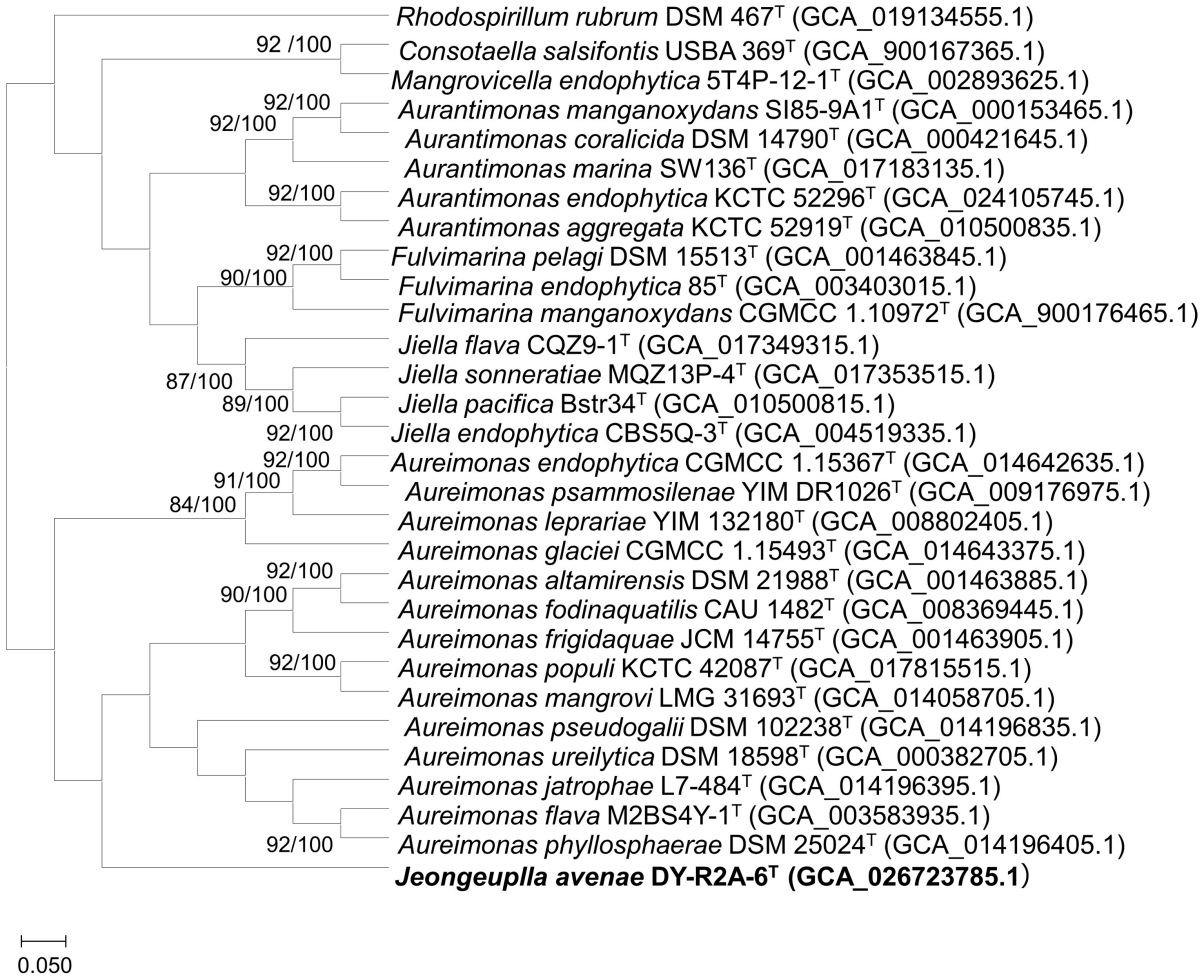
A phylogenomic tree of strain DY-R2A-6^T^ and other members of the family *Aurantimonadaceae* based on the up-to-date bacterial core gene set (UBCG) pipeline (concatenated alignment of 92 core genes). Gene support index (GSI, left) and bootstrap values (right) are indicated at the nodes. The genome sequence of *Rhodosirillum rubrum* DSM 467^T^ was used as outgroup. The scale bar corresponds to 0.050 substitutions per position. The 92 bacterial core genes were: *alaS*, *argS*, *aspS*, *cgtA*, *coaE*, *cysS*, *dnaA*, *dnaG*, *dnaX*, *engA*, *ffh*, *fmt*, *frr*, *ftsY*, *gmk*, *hisS*, *ileS*, *infB*, *infC*, *ksgA*, *lepA*, *leuS*, *ligA*, *nusA*, *nusG*, *pgk*, *pheS*, *pheT*, *prfA*, *pyrG*, *recA, rbfA*, *rnc*, *rplA*, *rplB*, *rplC*, *rplD*, *rplE*, *rplF*, *rplI*, *rplJ*, *rplK*, *rplL*, *rplM*, *rplN*, *rplO*, *rplP*, *rplQ*, *rplR*, *rplS, rplT, rplU, rplV, rplW, rplX, rpmA, rpmC, rpmI, rpoA, rpoB, rpoC, rpsB, rpsC, rpsD, rpsE, rpsF*, *rpsG, rpsH, rpsI, rpsJ, rpsK, rpsL, rpsM, rpsO, rpsP, rpsQ, rpsR, rpsS, rpsT, secA, secG, secY*, *serS*, *smpB, tig, tilS, truB, tsaD, tsf, uvrB, ybeY*, and *ychF*.

Whole or draft genome sequences of genera in the family *Aurantimonadaceae* were obtained from the National Center for Biotechnology Information (NCBI) database and their quality, completeness, and contamination were evaluated using the Microbial Genomes Atlas (MiGA) webserver (Rodriguez et al., 2018; Supplementary Table S1). To obtain accurate taxonomic positions, phylogenomic trees were calculated based on 92 core genes using the up-to-date bacterial core gene set (UBCG) pipeline with default parameters. The UBCG tree showed that strain DY-R2A-6^T^ formed a single cluster that was most closely related to the genus *Aureimonas* (Figure 2). However, the genus *Aureimonas* formed two branches, while the genera *Aurantimonas*, *Jiella*, *Fulvimarina, Consotaella*, and *Mangrovicella* all formed one branch. To obtain a high-resolution and refined taxonomic assignment for strain DY-R2A-6^T^ based on the whole-genome data, the Type Strain Genome Server (TYGS) was used for additional analyses on phylogenomic classifications. Consistent with that seen with the UBCG tree, the phylogenomic tree constructed using the TYGS also supported that strain DY-R2A-6^T^ formed a single branch and was most closely related to the genus *Aureimonas* (Supplementary Figure S2). The results of the phylogenomic analysis were consistent with strain DY-R2A-6^T^ representing a novel genus within the family *Aurantimonadaceae*.

To determine whether strain DY-R2A-6^T^ constituted a new species, we calculated the overall genome-related index (OGRI), which comprises digital DNA–DNA hybridization (dDDH), average nucleotide identity (ANI), and average amino acid identity (AAI) values, with closely-related strains (Chun et al., 2018). Our results indicated that the AAI between strain DY-R2A-6^T^ and closely related strains ranged from 61.2 to 64.2%, which was below the suggested threshold value of 80 to 85% for the classification of novel genera (Konstantinidis and Tiedje, 2005; Luo et al., 2014). Meanwhile, the ANI and dDDH values were 77.5 to 79.6% and 19.7 to 20.5%, respectively, for all closely-related strains (Supplementary Tables S2, S3). Additionally, the OrthoANI values obtained through the Orthologous Average Nucleotide Identity tool (OAT) were 72.7–75.1%, which supported the classification of the strain as a novel genus in the family *Aurantimonadaceae* (Supplementary Figure S3). As these values were considerably lower than the recommended dDDH (<70%), AAI (95–96%), and ANI (95%) cutoff values for bacterial species classification (Chun et al., 2018), the low OGRI values between the genome sequence of strain DY-R2A-6^T^ and closely genus were deemed insufficient to assign strain DY-R2A-6^T^ to the species level. Consequently, it is proposed that strain DY-R2A-6^T^ be recognized as a distinct taxon within the family *Aurantimonadaceae*, highlighting its novel classification. A circular map of the strain is shown in Supplementary Figure S4. Based on the results of our phylogenetic and phylogenomic analyses, we selected the type species of the genera *Jiella*, *Aurantimonas*, and *Aureimonas* as closely-related strains for chemotaxonomic study.

### 3.4 Genome annotation

The *de novo* assembly of the genome of strain DY-R2A-6^T^ using the Microbial Assembly application (version 8.0, accession number: CP113520.1– CP113521.1) revealed a single circular chromosome with a length of 5,909,138 bp and a plasmid with a length of 20,172 bp. The genome was assessed for quality using the Microbial Genomes Atlas (MiGA) web server, with the results indicating a completeness of 100%, a contamination rate of 1.9%, and a quality score of 90.5% (Supplementary Table S1). The depth of sequencing coverage was 256× after assembly combined the sequence obtained on PacBio Sequel System and the Illumina platform. The G + C content of the genome was 69.1%, which aligns with the G + C content reported for other genera within the family *Aurantimonadaceae* (Denner et al., 2003; Ren et al., 2014; Liang et al., 2015; Zhang K. et al., 2020). Gene annotation using the Prokaryotic Genome Annotation Pipeline (PGAP) from the NCBI database identified a total of 5,558 protein-coding genes and 69 RNA genes, including four 5S rRNA genes, four 16S rRNA genes, four 23S rRNA genes, four ncRNAs, and 53 rRNA genes. Analysis of the cluster orthologous group (COG) annotations revealed that most of the assigned COGs were classified as unknown (30.7%), followed by amino acid transport and metabolism (8.4%), and inorganic ion transport and metabolism (6.8%) (Supplementary Figures S4, S5).

### 3.5 Genome-derived features of strain DY-R2A-6^T^

To identify key metabolites, we utilized the BlastKOALA pipeline and RAST seed to reconstruct metabolic pathways based on the genome sequence of strain DY-R2A-6^T^. The following sections delineate all the metabolites predicted using this approach.

(i) Phenotypic and biochemical characteristics

Cell motility: Fourteen genes associated with bacterial chemotaxis and 32 genes linked to flagellar assembly were identified. Bacterial chemotaxis allows cells to move toward distant sources of food (Bray, 2002) and is facilitated by a variety of proteins, including chemotaxis protein CheD [EC:3.5.1.44]; chemotaxis protein methyltransferase CheR [EC:2.1.1.80]; chemotaxis protein methyltransferase CheR [EC:2.1.1.80]; two-component system, chemotaxis family, protein-glutamate methylesterase/glutaminase CheB [EC:3.1.1.61 3.5.1.44]; two-component system, chemotaxis family, sensor kinase CheA [EC:2.7.13.3]; purine-binding chemotaxis protein CheW; and two-component system, chemotaxis family, chemotaxis protein CheY. Additionally, flagellar motility-related genes, such as those encoding the hook-basal body flagellin protein FlaA; the flagellar motor switch protein FliM; the basal body rod proteins FlgB, FlgC, FlgF, and FlgG; the flagellar basal-body rod modification protein FlgD; the flagellar hook protein FlgE; the flagellar L-ring protein FlgH; the flagellar motor switch protein FliN; and the flagellar P-ring protein FlgI, were also found in the genome. However, the absence of genes coding for flagellar biosynthesis and flagellar motor rotation proteins, as well as flagellum-specific ATP synthase-related genes, may account for the weak motility of strain DY-R2A-6^T^.

Fatty acid metabolism: The entire pathway for lipid metabolism, which encompasses fatty acid metabolism, was reconstructed from the genome. The pathway included six genes for fatty acid biosynthesis initiation (accA, accB, accC, accD, fabD, fabH), six for elongation (fabB, fabF, fabG, fabZ, fabA, fabI), one for acyl-CoA synthesis (fadD), and five for β-oxidation (fadA, fadE, fadJ, paaF, acd).

Polar lipid metabolism: The biosynthetic pathway for the polar lipids phosphatidylcholine and phosphatidylethanolamine was also reconstructed from the genome of strain DY-R2A-6^T^. Enzymes involved in phosphatidylcholine biosynthesis, such as phosphatidylethanolamine/phosphatidyl-N-methylethanolamine N-methyltransferase [EC:2.1.1.17 2.1.1.71], as well as those involved in phosphatidylethanolamine biosynthesis, including phosphatidate cytidylyltransferase [EC:2.7.7.41], CDP-diacylglycerol–serine O-phosphatidyltransferase [EC:2.7.8.8], and phosphatidylserine decarboxylase [EC:4.1.1.65], were also annotated.

Stress response: A total of 62 stress response-related genes were detected in the genome of strain DY-R2A-6^T^, including genes associated with osmotic stress (17), oxidative stress (34), detoxification (9), no subcategory stress response (4), periplasmic stress (3), and no subcategory stress response (4). Osmotic stress-related genes involved in osmoregulation, osmoprotectant ABC transporter YehZYXW of Enterobacteriales, and the synthesis of osmoregulated periplasmic glucans, choline, and betain uptake, as well as betain biosynthesis, were observed. The genome also contained genes related to oxidative stress, such as those involved in glutathione biosynthesis, the gamma-glutamyl cycle, and the non-redox cycle, as well as genes encoding glutaredoxins. Detaxification-related genes included those associated with the uptake of selenite and the glutathione-dependent formaldehyde detoxification pathway. Periplasmic stress-related genes involved in the periplasmic stress response and genes associated with no subcategory stress response, including dimethylarginine metabolism and the hfl operon, were also identified.

(ii) Analysis of gene clusters involved in metabolite biosynthesis

Using antiSMASH, the whole genome of strain DY-R2A-6^T^ was annotated for the identification of natural products (Blin et al., 2021), as these could have the potential for a variety of applications. Eight BGCs were found (Supplementary Table S4), including bacillomycin D, carotenoids, and cittilin A/cittilin B, as well as some unknown clusters. Bacillomycins constitute a group of antifungal polypeptide antibiotics isolated from *Bacillus subtilis* that are well-known for their ability to inhibit the growth of pathogenic fungi such as *Malassezia globose* (Wu et al., 2020), *Fusarium graminearum* (Gu et al., 2017), and *Candida* spp. (Tabbene et al., 2015). The presence of bacillomycins suggests that strain DY-R2A-6^T^ may contribute to the antifungal activity of its host. Carotenoids, also known as tetraterpenoids, are produced by many plants, algae, and bacteria, and play an essential role in biological oxygenation. Carotenoids are highly effective antioxidants with roles in ROS scavenging and defense against oxidative stress in plants (Zuluaga et al., 2017; Shen et al., 2018). Through their antioxidant properties, carotenoids contribute to the promotion of plant growth under various environmental stresses. Cittilin A is a natural product found in *Myxococcus xanthus* that has potential biological functions as an inhibitor of carbon storage regulator A (CsrA) (Hug et al., 2020). To the best of our knowledge, this is the first description of a BGC gene cluster in the family *Aurantimonadaceae*. Our findings provide valuable insight into these natural products and their potential applications in plant growth.

### 3.6 Chemotaxonomic characterization

The major cellular fatty acids in strain DY-R2A-6^T^ were C_16:0_ (11.3%), C_19:0_ cyclo ω8c (13.7%), and summed feature 8 (C_18:1_ω7c or/and C_18:1_ω6c) (72.8%) (Table 2). The most closely related species in the genera *Jiella*, *Aurantimona*s, and *Aureimonas* also contained summed feature 8 as the most abundant cellular fatty acids. However, strain DY-R2A-6^T^ and *Aureimonas* also contained C_16:0_ as one of the most abundant fatty acids, which differentiates them from *Jiella* and *Aurantimonas*. Additionally, strain DY-R2A-6^T^ and *Jiella* contained C_19:0_ cyclo ω8c, which distinguishes them from genus *Aurantimonas* and *Aureimonas*. Strain DY-R2A-6^T^ had a more diverse major fatty acid profile compared with closely related strains. As with other genera within the family *Aurantimonadacea*, ubiquinone-10 was the predominant respiratory quinone in strain DY-R2A-6^T^. The major polar lipids identified in strain DY-R2A-6^T^ were diphosphatidylglycerol, phosphatidylethanolamine, phosphatidylmonomethylethanolamine, phosphatidylglycerol, phosphatidylcholine, one unknown aminolipid, and four unknown polar lipids (Supplementary Figure S6). The polar lipid profile of strain DY-R2A-6^T^ was distinguished from that of the most closely related strains by the absence of one phospholipid.

**Table 2:** Cellular fatty acid profiles (>1%) of strain DY-R2A-6^T^ and those of its closely-related species.

Fatty acid	1	2	3	4
C_16:0_	**11.3**	4.0	**15.4**	5.3
C_18:0_	2.2	3.7	1.1	1.5
C_15:0-_anteiso	/	/	1.2	0.3
C_19:0_ cyclo ω8c	**13.7**	7.3	7.6	**13.7**
3OH-C_18:0_	/	0.9	2.5	0.7
2OH-C_18:1_	/	/	/	4.3
Summed feature 2*	/	2.5	0.7	0.7
Summed feature 3*	/	/	2.7	/
Summed feature 8*	**72.8**	**81.7**	**67.9**	**73.6**

Strains: (1) *Jeongeuplla avenae* DY-R2A-6^T^; (2) *Aurantimonas aggregata* KCTC 52919^T^; (3) *Aureimonas frigidaquae* KCTC 12893^T^; (4) *Jiella aquimaris* JCM 30119^T^. All the data were obtained in the present study.

Major components are in bold; /, not detected.

*Summed features of fatty acid profiles: grouping of two or three fatty acids that cannot be separated by gas–liquid chromatography (GLC) with the MIDI System. Summed feature 2 includes C_12:0_ aldehyde/unknown (10.9); summed feature 3 includes C_16:1_ ω6c and/or C_16:1_ ω7c; and summed feature 8 is listed as C_18:1_ ω7c and/or C_18:1_ ω6c.

Taken together, the phenotypic, physiological, phylogenetic, and biochemical results support that strain DY-R2A-6^T^ belongs to the family *Aurantimonadaceae*. However, the low 16S rRNA gene sequence similarity (≤96.3%) and the low ANI (<79.6%), AAI (<75.1%), and dDDH (<20.5%) values suggest that strain DY-R2A-6^T^ differs from its most closely related strains. Additionally, phylogenetic trees based on the 16S rRNA gene and core gene sets from the whole-genome sequence indicated that strain DY-R2A-6^T^ formed a single branch within the family *Aurantimonadaceae*, implying that strain DY-R2A-6^T^ represents a novel genus within this family.

### 3.7 Identification of carotenoid pigments

**Figure 3:**
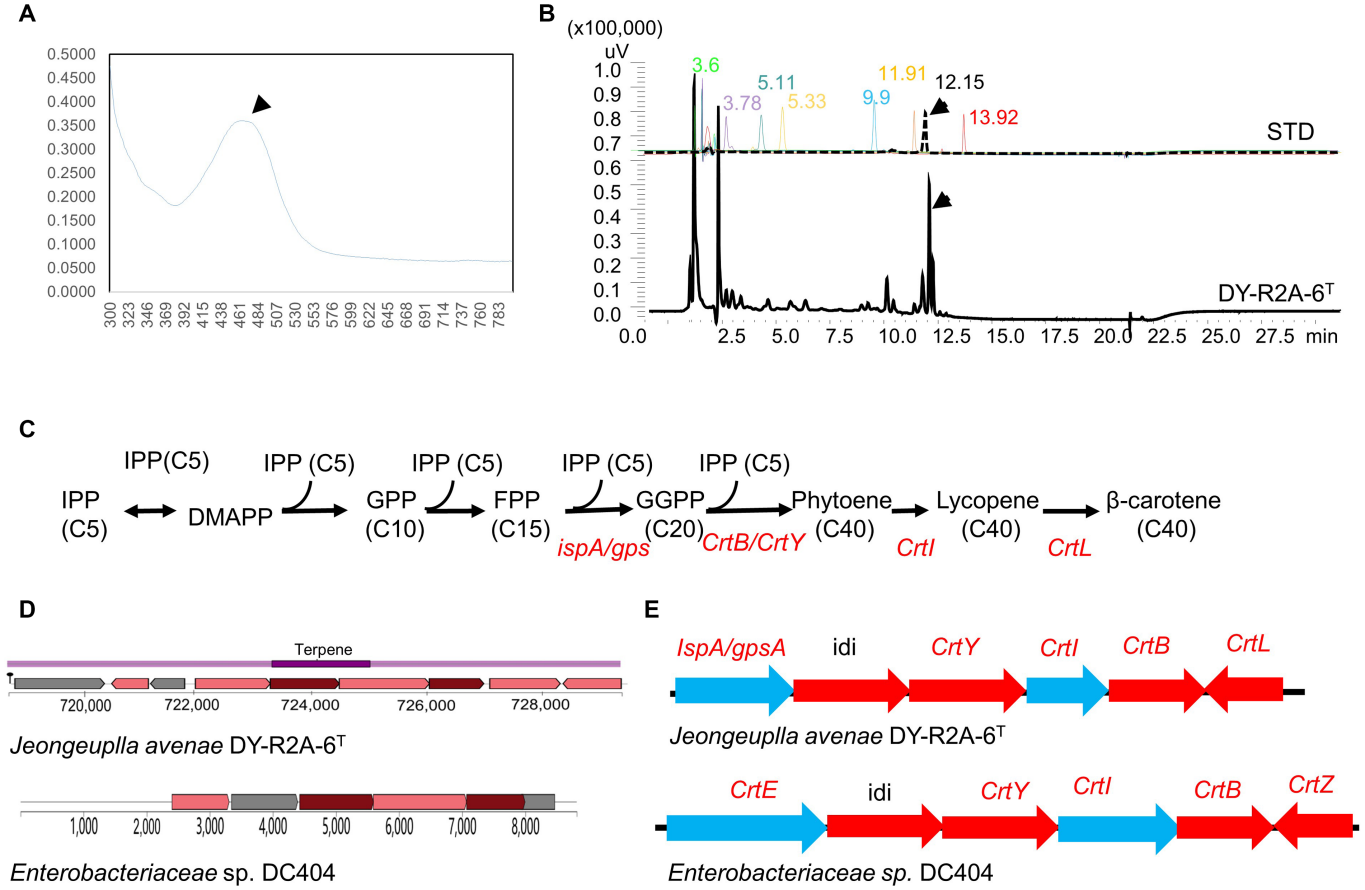
Identification of carotenoids in strain DY-R2A-6^T^. **(A)** UV–visible absorption spectra of the major carotenoids of strain DY-R2A-6^T^. **(B)** Chromatogram of carotenoid standard solutions and the carotenoid of strain DY-R2A-6^T^; the identified compounds included neoxanthin (RT 3.6), violaxanthin (RT 3.78), zeaxanthin (RT 5.11), lutein (RT 5.33), cryptoxanthin (RT 9.9), α-carotene (RT 11.91), β-carotene (RT 12.15), and lycopene (RT 13.92). **(C)** Carotenogenic gene clusters and the proposed β-carotene biosynthetic pathway in strain DY-R2A-6^T^. **(D)** A comparison of the predicted β-carotene biosynthesis gene cluster of strain DY-R2A-6^T^ (top) with that of the most similar cluster (*Enterobacteriaceae* sp. DC404) (bottom) using antiSMASH. **(E)** The β-carotene biosynthesis pathway in strain DY-R2A-6^T^ involves IPP (isopentenyl pyrophosphate), DMAPP (dimethylallyl pyrophosphate), FPP (farnesyl diphosphate), GGPP (geranylgeranyl diphosphate), *crtE* (GGPP synthase), *crtB* (phytoene synthase), *crtI* (phytoene desaturase/dehydrogenase), *crtY* (lycopene cyclase), *crtZ* (β-carotene hydroxylase), *idi* (isopentenyl-diphosphate delta-isomerase), and *crtL* (lycopene beta cyclase).

Strain DY-R2A-6^T^ was found to produce a creamy pink to yellowish pigment when grown on R2A medium for 3 days, indicative of carotenoid accumulation. Initially, we employed the genome mining tool antiSMASH to predict the potential carotenoid type based on related BGCs. The results suggested that strain DY-R2A-6^T^ had a carotenoid BGC similar to that of *Enterobacteriaceae* sp. DC404, which contained the genes necessary for zeaxanthin synthesis but lacked the zeaxanthin glucoside synthesis gene crtX (Sedkova et al., 2005). These results suggested that the pink to yellow pigment was either zeaxanthin or an upstream/downstream product. The pigment was extracted using methanol and analyzed using UV–Vis absorption spectroscopy, and showed one absorption peak at 465 nm (Figure 3A). The spectrum of strain DY-R2A-6^T^ closely matched those of β-carotene extracted from olive oil (λmax values at 462 nm) (Domenici et al., 2014), carrots, and fruits (λmax values at 461 nm) (Karnjanawipagul et al., 2010; Sricharoen et al., 2016), and completely matched with β-carotene (λmax values at 465 nm) (Chabera et al., 2009).

Several common carotenoid standard samples, including HPLC-grade zeaxanthin, lutein, cryptoxanthin, β-carotene, α-carotene, violaxanthin, and neoxanthin, were simultaneously analyzed via HPLC (Figure 3B). The results revealed that the pigment extracted from strain DY-R2A-6^T^ had a main peak with a retention time of 12.17 min, which corresponds to the peak produced by the authentic β-carotene standard (12.15 min) (Figure 3B). Based on the above results, the pigment of strain DY-R2A-6^T^ was identified as β-carotene.

### 3.8 The β-carotene biosynthesis pathway

Carotenoids are colored tetraterpene pigments synthesized by a variety of organisms, including plants, bacteria, and algae. Carotenoid synthesis occurs via the mevalonate (MVA) and the 2-C-methyl-D-erythritol 4-phosphate (MEP) pathways, which generate isopentenyl diphosphate (IPP) and its allylic isomer dimethylallyl diphosphate (DMAPP), essential carotenoid precursors.

Although enhancing carotenoid production through metabolic engineering has been achieved in microalgae and bacteria, and some carotenoid synthesis enzymes have been cloned (Sedkova et al., 2005; Siddaramappa et al., 2018; Zhang J. et al., 2020; Chen et al., 2023), further research is needed to fully understand the carotenoid biosynthesis pathway and the modifications of the final structure. Accordingly, we next annotated the complete genome of strain DY-R2A-6^T^ using PGAP, BlastKOALA, and antiSMASH to characterize the carotenoid biosynthesis pathway in this bacterium. The results revealed that IPP was synthesized through the MEP pathway, and several enzymes involved in the MEP pathway were predicted to be encoded in the genome of strain DY-R2A-6^T^, including 1-deoxy-D-xylulose-5-phosphate synthase [EC:2.2.1.7] (encoded by dxs), 1-deoxy-D-xylulose-5-phosphate reductoisomerase [EC:1.1.1.267] (dxr), intracellular septation protein A (ispZ), 4-hydroxy-3-methylbut-2-enyl diphosphate [EC:1.17.7.4] (ispH), 4-hydroxy-3-methylbut-2-enyl diphosphate synthase (flavodoxin) [EC:1.17.7.1] (ispG), phosphoenolpyruvate carboxykinase [EC:4.1.1.49] (pckA), and pyruvate kinase [EC:2.7.1.40] (pyk). These findings are consistent with previous studies showing that the MEP pathway is typically found in most bacteria and plant plastids and the MVA pathway is mainly present in eukaryotes and archaea (Paniagua-Michel et al., 2012; Boronat and Rodriguez-Concepcion, 2015).

Through whole-genome annotation and gene comparison, several genes of strain DY-R2A-6^T^ were found to be directly involved in the synthesis of carotenoids. Subsequently, the composition of the carotenoid biosynthesis pathway of strain DY-R2A-6^T^ was then predicted based on the identified gene cluster (Figures 3C–E). Like Enterobacteriaceae sp. DC404 (Sedkova et al., 2005), the first step involves IPP (C5) isomerization to geranyl PP (GPP, C10) and farnesyl PP (FPP, C15), which then undergo further isomerization to GGPP through FPP synthase (ispA), PSase (crtB), or GspSA (gsp) (GGPPS [CrtE] for Enterobacteriaceae sp. DC404). Next, a single enzyme, PSase (crtB), mediates the head-to-head coupling of two GGPP molecules to form phytoene, which then undergoes four desaturation steps catalyzed by the enzyme lycopene beta cyclase (encoded by crtl), yielding lycopene. Four double bonds are introduced into phytoene to produce lycopene, which then serves as a precursor for the production of β-carotene (crtL), zeaxanthin (crtZ), and astaxanthin (crtW) (Kim S. H. et al., 2014; Lee et al., 2018). The whole-genome annotation results confirmed that strain DY-R2A-6^T^ was a producer of β-carotene, which was consistent with the HPLC results (Figure 3B).

### 3.9 The impact of strain DY-R2A-6^T^ on plant growth under saline conditions

Analysis of the genome of strain DY-R2A-6^T^ revealed the presence of a carotenoid BGC and the extracted pigment was identified as β-carotene by UV–vis and HPLC. In plants that produce them, carotenoids reduce oxidative stress by scavenging abiotic stress-related ROS. Additionally, it has been reported that plants mitigate the effects of environmental stress partly by increasing β-carotene production. However, studies focusing on the role of β-carotene-producing bacteria on environmental stress in plants are scarce.

**Figure 4:**
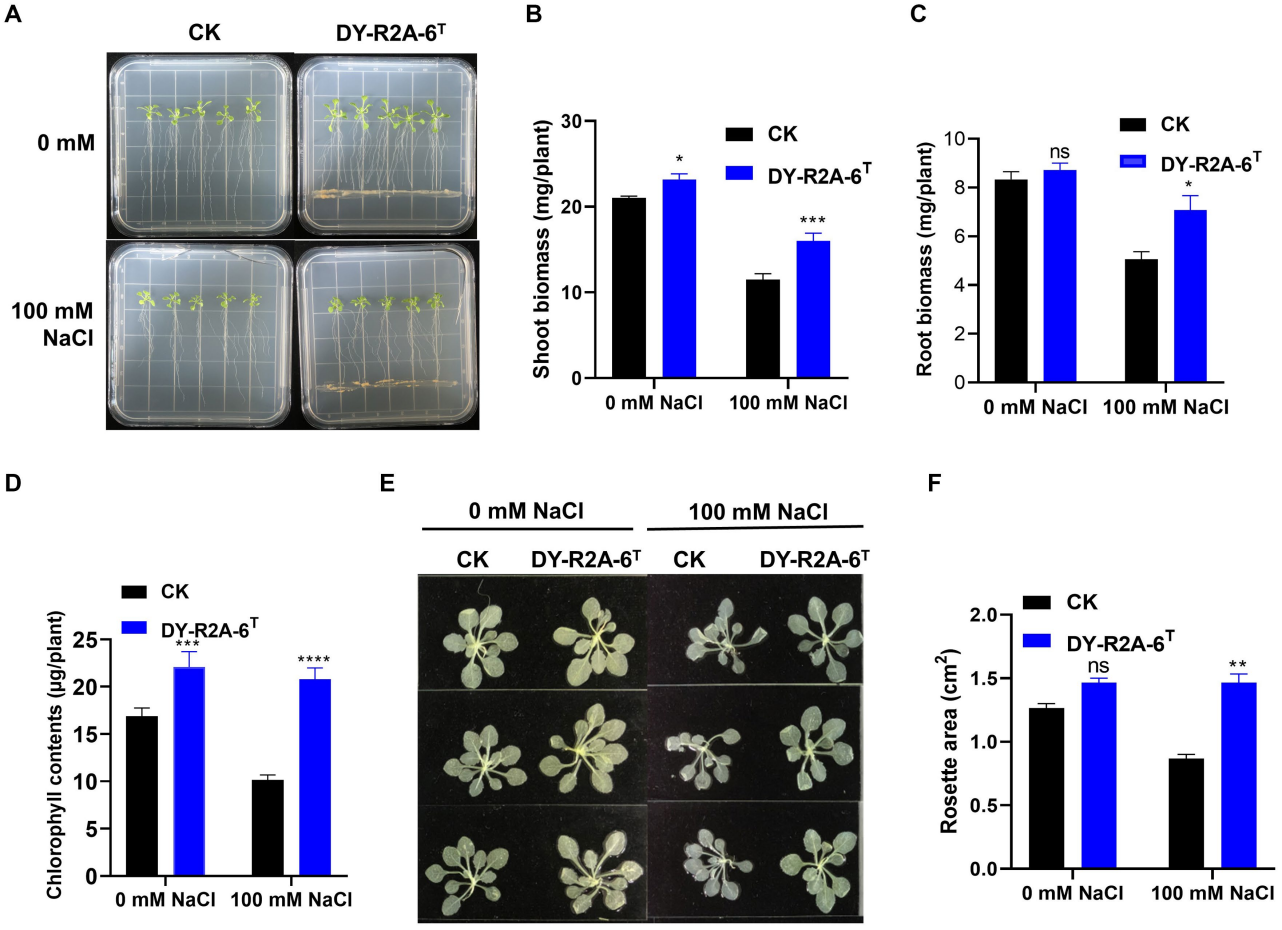
Effects of cultivation with strain DY-R2A-6^T^ on the growth of Arabidopsis seedlings under salt stress conditions. **(A)** Representative image of seedlings grown by strain DY-R2A-6^T^ for 10 days in ½ MS medium supplemented with NaCl (100 mM) at 22°C. **(B,C)** Quantification of shoot **(B)** and root **(C)** biomass production after 10 days by strain DY-R2A-6^T^ under salinity condition (100 mM NaCl). Data represent the mean fresh weight ± SD of at least 18 seedlings from three different plates. **(D)** Measurement of seedling total chlorophyll contents. **(E)** Representative images of seedlings decolorized in 100% ethanol. **(F)** Measurement of the rosette area of Arabidopsis seedlings in panel **(E)** using ImageJ software. *p < 0.05, **p < 0.01, and ***p < 0.001, two-way ANOVA. Error bars represent the standard deviation of the mean (*n* = 18 for seedling biomass and *n* = 9 for leaf area).

Strain DY-R2A-6^T^ was cultivated with 7-day-old *Arabidopsis* seedlings to evaluate its potential for promoting plant growth under normal (0 mM NaCl) or salt stress (100 mM NaCl) conditions. After 10 days, shoot biomass, root biomass, rosette area, and total chlorophyll contents were measured (Figures 4A–E). The results showed that cultivation with strain DY-R2A-6^T^ increased shoot biomass under both normal and salt stress conditions (by 1.1- and 1.4-fold, respectively). Cultivation with strain DY-R2A-6^T^ also increased the rosette area and the chlorophyll content under the salt stress (Figures 4B–F). Additionally, in the presence of strain DY-R2A-6^T^, root biomass under salt stress conditions was also significantly higher (1.39-fold) than that seen in the controls; however, no difference in root biomass was observed under normal conditions (Figure 4D).

Studies have shown that osmotic/salinity conditions can markedly decrease auxin levels in the roots and stems of *Arabidopsis* plants (Chabera et al., 2009; Zhang et al., 2012; Chen et al., 2023). To explore whether the DY-R2A-6^T^-induced increase in root biomass observed under salt conditions was due to the inhibition of a reduction in auxin levels, we employed Arabidopsis transgenic lines expressing the DR5::GUS auxin reporter. As previously demonstrated, we observed that salt treatment led to a substantial reduction in the GUS signal in the roots (Iglesias et al., 2014; Ding et al., 2015; Liu et al., 2015); however, no such reduction in GUS levels was observed in the presence of strain DY-R2A-6^T^ (Supplementary Figure S7).

**Figure 5:**
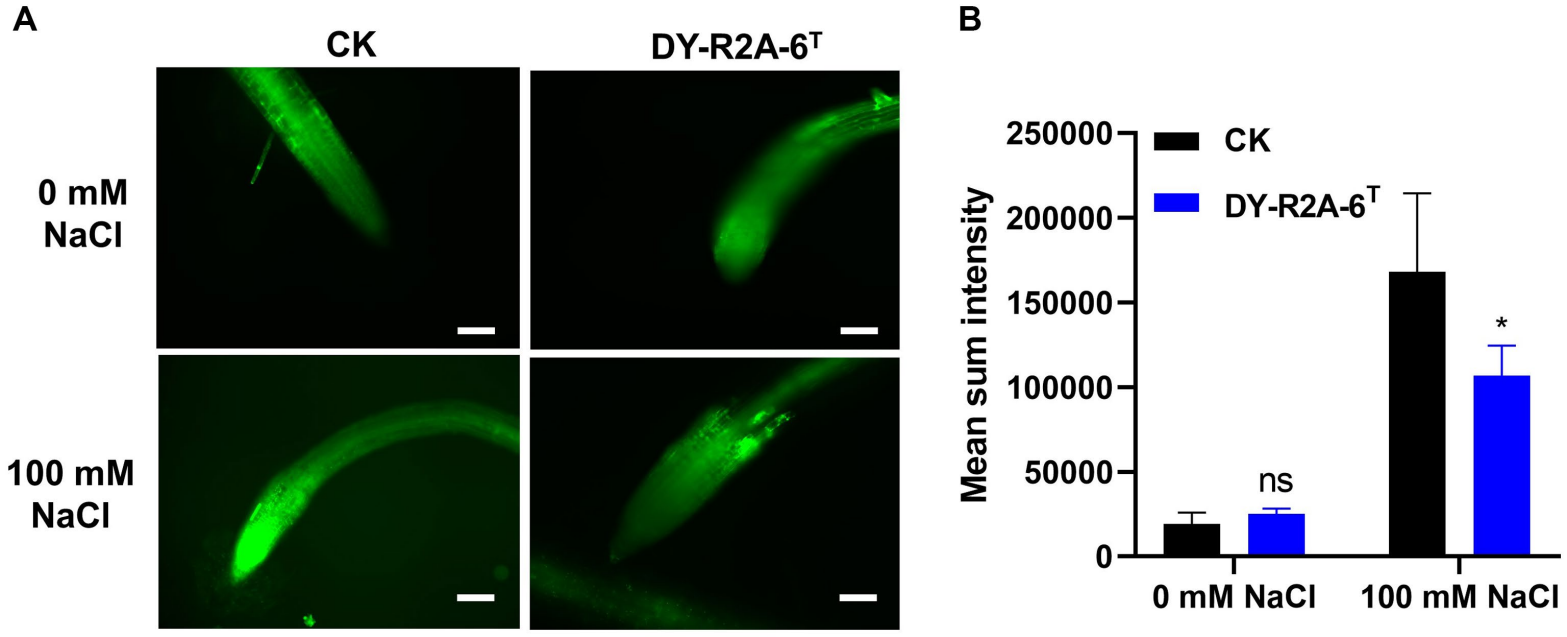
Assessment of intercellular reactive oxygen species (ROS) in the roots using 2′,7′–dichlorodihydrofluorescein diacetate (DCFH-DA) staining. **(A)** ROS detection via DCFH-DA staining by strain DY-R2A-6^T^ under salt stress conditions (100 mM NaCl). Scale bars: 100 μm. **(B)** Measurement of fluorescence intensity using ImageJ software. *p < 0.05, two-way ANOVA. Error bars represent the standard deviation of the mean (*n* = 3).

**Figure 6:**
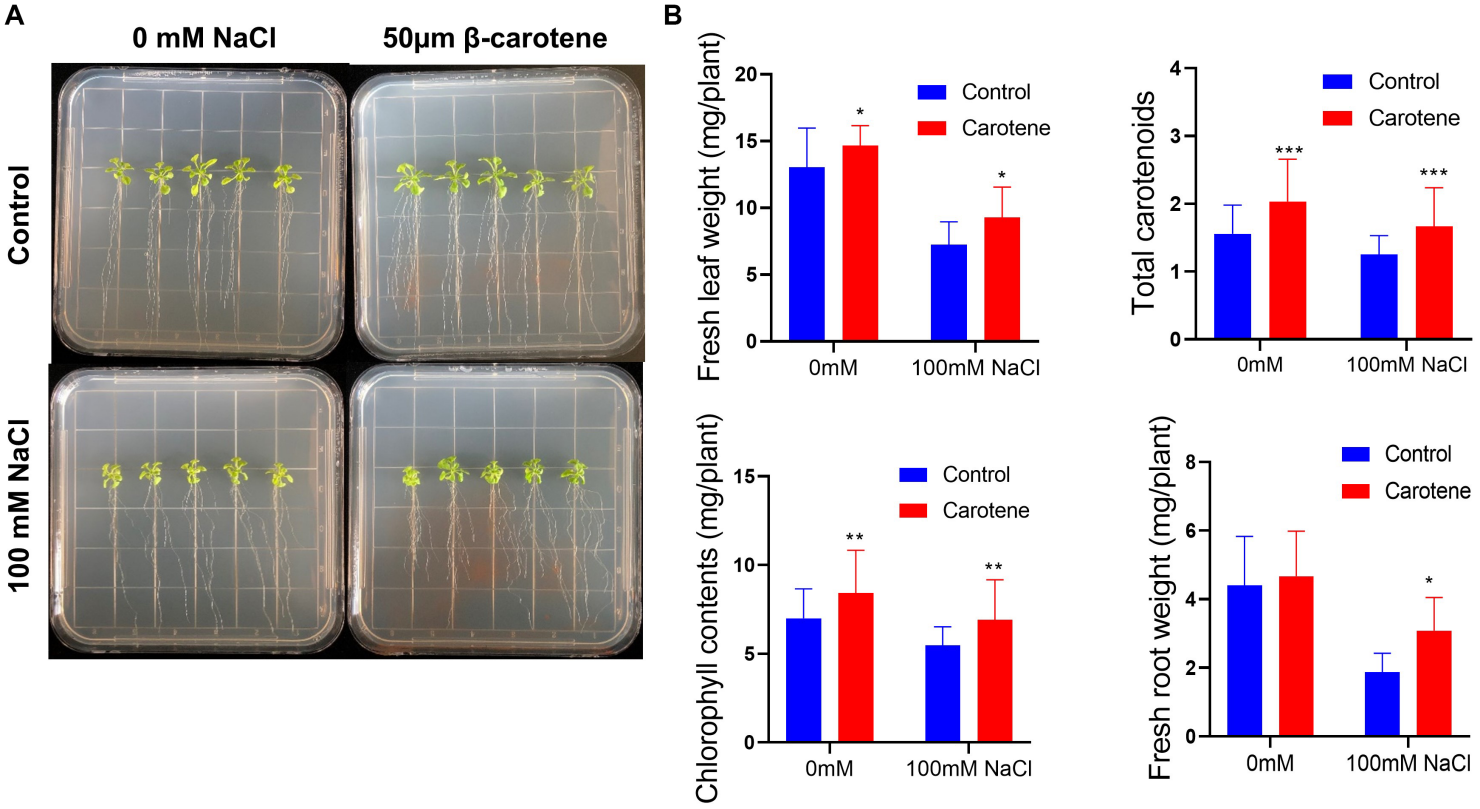
Effects of β-carotene under salinity conditions. **(A)** Representative image of seedlings by 50 μmol β-carotene spray treatment under salinity conditions. **(B)** Evaluation of total fresh leaf weight, total carotenoids, total chlorophyll contents, and fresh root weight. *p < 0.05, **p < 0.01, and ***p < 0.001, as determined by two-way ANOVA. Error bars indicate the standard deviation of the mean (*n* = 18).

The growth of plants under salt conditions is adversely affected by ionic and osmotic stress, along with nutrient imbalance. This environment promotes the generation of free oxygen radicals, causing an overabundance of ROS. These ROS are highly reactive and toxic, inflicting damage to vital components such as lipids, proteins, and DNA, consequently inducing oxidative stress within plant cells. The excessive accumulation of ROS disrupts the metabolic processes within plant cells, leading to detrimental effects on cell organelles and the tissues of both shoots and roots. 2′,7′-dichlorodihydrofluorescein diacetate (DCFH-DA) staining can provide a qualitative estimate of ROS generation and information on ROS localization in root tips. Here, we found that ROS levels in the roots of *Arabidopsis* were markedly higher under salt treatment than under normal conditions; however, cultivation with strain DY-R2A-6^T^ mitigated the effects of salinity, but no such reduction in ROS fluorescence was observed under normal conditions (Figure 5A). Next, we quantified the relative fluorescence intensity by measuring the mean of the sum intensity of at least three roots using ImageJ software. We found that the fluorescence intensity was considerably diminished by cultivation with strain DY-R2A-6^T^ under salt stress, and the fluorescence intensity was similar under normal conditions (Figure 5B). These results suggested that strain DY-R2A-6^T^ mitigates the effects of salt stress on plants by scavenging the resultant ROS. To confirm that the mitigation of salinity stress in Arabidopsis seedlings by strain DY-R2A-6^T^ could be attributed to the β-carotene produced by the strain, we investigated the impact of externally applied β-carotene under salinity condition. Specifically we sprayed the roots of 7-day-old seedlings with 50 μM of β-carotene and allowed them to incubate for an additional 10 days, we observed partially enhanced plant growth under both normal and salinity conditions, as shown in Figure 6.

Similar previous studies showed that, when externally applied via foliar spray, β-carotene can alleviate salt stress by reducing the amount of hydrogen peroxide (H_2_O_2_) which can increase the ROS contents (Babaei et al., 2022). Additionally, bacteria that produce nostoxanthin have been found to reduce ROS-induced stress in plants and facilitate plant growth when exposed to salinity stress (Jiang et al., 2023). β-carotene is a member of the carotenoid family, a well-known group of isoprenoid pigments extensively researched for their ability to quench ROS and plant self-defense mechanisms, ultimately mitigating oxidative stress-induced damage (Li et al., 2019). Carotenoids, including zeaxanthin, have been shown to reduce ROS levels, possibly by inducing lipid peroxidation and actively scavenging free radicals. This effect may be attributed to the potential production of ROS-scavenging enzymes by bacteria, such as APX, CAT, SOD, NOX, GR, PRX, and GST enzymes, which play pivotal roles in the physiological response to oxidative stress. Furthermore, carotenoid intake, such as nostoxanthin, has been linked to a reduced incidence of certain chronic diseases by minimizing *in vivo* oxidative damage caused by ROS and nitrogen species (RNS). Carotenoids are recognized as effective singlet oxygen quenchers with robust antioxidant properties (Di Mascio et al., 1989).

In this study, we found the important discovery of a new genus and species belonging to the family *Aurantimonadaceae*, which we propose to name *Jeongeuplla avenae* gen. nov. and sp. nov. By analyzing the cluster of carotenoid biosynthesis genes in the genome, we were able to predict the type of pigment produced by strain DY-R2A-6^T^, which we subsequently confirmed as β-carotene using UV–vis and HPLC analysis. We also found that this novel β-carotene-producing bacterium significantly enhances the tolerance of Arabidopsis seedlings to salinity stress through ROS scavenging. We further confirmed that spraying the plants with β-carotene under saline conditions partially increased their biomass. Nevertheless, it is essential to emphasize the need for further investigations to understand the complex mechanisms underlying salt-induced ROS scavenging by microbes in plants. Such studies have the potential to advance our knowledge of how microbes contribute to stress resilience in plants.

## 4 Description and conclusion

In this study, we isolated a novel strain from oat seeds, which we designated DY-R2A-6^T^. Using a polyphasic taxonomic approach, which included phenotypic, physiological, phylogenomic, and biochemical analysis, we confirmed that strain DY-R2A-6^T^ is a member of the family *Aurantimonadaceae*. Phylogenetic analysis based on the 16S rRNA gene sequence and core gene sets from the whole-genome sequence revealed that strain DY-R2A-6^T^ forms a distinct lineage within this family. Genomic relatedness, as determined by AAI, ANI, and dDDH values, further confirmed that DY-R2A-6^T^ differs from its phylogenetically closest genera. Collectively, these findings support the inclusion of DY-R2A-6^T^ as a new member of the family *Aurantimonadaceae*. Strain DY-R2A-6^T^ can producing a pink-yellow pigment, it was identified as β-carotene. Furthermore, it has been established that β-carotene plays a crucial role in mitigating oxidative stress by ROS associated with abiotic stress. A detailed description is provided below.

### 4.1 Description of *Jeongeuplla* gen. nov.

Jeongeuplla (Jeon.geup’l’l.a. N.L. fem. n. Jeongeuplla, named after Jeongeup, a Korean city, the main oat seed-growing area in Korea).

The cells exhibit Gram-negative staining behavior, are short rod-shaped or ovoid, and flagella. They are catalase- and oxidase-positive and colonies grown on R2A medium produce a pink to yellowish pigment. The major cellular fatty acids identified are C_16:0_, C_19:0_ cyclo ω8c, and summed feature 8. The predominant respiratory quinone is ubiquinone-10. The major polar lipids detected are diphosphatidylglycerol, phosphatidylethanolamine, phosphatidylmonomethylethanolamine, phosphatidylglycerol, phosphatidylcholine, one unknown aminolipid, and four unknown polar lipids. The member of this genus constituted a distinct branch within the 16S rRNA gene- and the 92 core gene-based phylogenomic trees. The genus *Jeongeuplla* is a member of the family *Aurantimonadaceae*, order *Rhizobiales*, and the type species is *Jeongeuplla avenae*.

### 4.2 Description of *Jeongeuplla avenae* sp. nov.

Jeongeuplla avenae (a.ve’nae. L. fem. n. avena, genus of plants; L. gen. fem. n. avenae, of *Avena sativa*).

Cells are creamy pink to yellowish and form circular, smooth, opaque colonies (1–4 mm in diameter) when grown on R2A medium for 3 days. Cells are short rod-shaped or ovoid, weakly motile, Gram-negative, and flagella. The cell width ranges from 0.9 to 1.1 μm and the cell length ranges from 0.9 to 2.2 μm. The optimal growth conditions for the strain are temperatures between 10 and 30°C, a pH between 6 and 10, and a NaCl concentration of 0 to 2.5%. Strain DY-R2A-6^T^ is positive for catalase and oxidase activity. Additionally, it demonstrates positivity for the enzymes alkaline phosphate, esterase (C4), esterase lipase (C8), leucine arylamidase, valine arylamidase, cystine arylamidase, trypsin, acid phosphate, and naphthol-AS-BI-phosphohydrolase on API ZYM strips. Positivity for urease hydrolysis, esculin hydrolysis, glucose assimilation, and arabinose assimilation are observed on API 20NE strips, while all other reactions are negative. The strain exhibits positive reactions for several substrates on API 50CH strips, including L-arabinose, D-ribose, D-xylose, methyl-β,D-xylopyranoside, D-adonitol, D-galactose, D-glucose, D-fructose, D-mannose, L-rhamnose, D-mannitol, and D-fucose, and negativity for all other substrates. The major fatty acids are C_16:0_, C_19:0_ cyclo ω8c, and summed feature 8. Ubiquinone-10 is the predominant respiratory quinone. The major polar lipids identified were diphosphatidylglycerol, phosphatidylethanolamine, phosphatidylmonomethylethanolamine, phosphatidylglycerol, phosphatidylcholine, one unknown aminolipid, and four unknown polar lipids.

*Jeongeuplla avenae* DY-R2A-6^T^ (= KCTC 82985^T^ = GDMCC 1.3014^T^) was designated as the type strain and was isolated from oat seeds grown in Jeongeup, Korea. The 16S rRNA and whole genome accession numbers for the strain are OL709414.1 and CP113520.1–CP113521.1, respectively.

## Data availability statement

The datasets presented in this study can be found in online repositories. The names of the repository/repositories and accession number(s) can be found in the article/Supplementary material.

## Author contributions

LJ: Data curation, Investigation, Methodology, Writing – original draft. YP: Resources, Writing – review & editing. K-HK: Methodology, Visualization, Writing – review & editing. DJ: Resources, Writing – review & editing. HC: Methodology, Visualization, Writing – review & editing. A-RH: Resources, Writing – review & editing. CK: Funding acquisition, Writing – review & editing. JL: Conceptualization, Funding acquisition, Project administration, Resources, Supervision, Writing – review & editing.

## Footnotes

^1^ http://carotenoiddb.jp/index.html

^2^ https://lpsn.dsmz.de/family/aurantimonadaceae

^3^ https://dna.macrogen.com/

^4^ https://blast.ncbi.nlm.nih.gov/Blast.cgi

^5^ http://www.ezbiocloud.net

^6^ http://ggdc.dsmz.de/distcalc2.php

^7^ http://enve-omics.ce.gatech.edu/aai/

^8^ http://enve-omics.ce.gatech.edu/ani/

^9^ https://cgview.ca/

^10^ https://antiSMASHsecondarymetabolites.org

^11^ https://tygs.dsmz.de/

^12^ https://blog.naver.com/ezmass
