# Supplementary material for: Jeongeuplla avenae gen. nov., sp. nov., a novel β-carotene-producing bacterium that alleviates salinity stress in Arabidopsis
Source: Front Microbiol. 2023 Dec 6;14:1265308. doi: 10.3389/fmicb.2023.1265308 (PMC10731981; doi:10.3389/fmicb.2023.1265308)
Supplement: Supplementary file 1 [file Data_Sheet_1.pdf]

Supplementary Information

Jeongeuplla avenae gen. nov., sp. nov., a Novel β-Carotene-producing Bacterium that Alleviates Salinity Stress in Arabidopsis

Lingmin Jiang[1,4], Yuxin Peng[1], Ki-Hyun Kim[1], Doeun Jeon[1], Hanna Choe[1], Ah-Reum Han[2], Cha Young Kim[1], Jiyoung Lee[1,3,*]

[1] Biological Resource Center, Korean Collection for Type Cultures (KCTC), Korea Research Institute of Bioscience and Biotechnology (KRIBB), Jeongeup 56212, Republic of Korea

[2] Advanced Radiation Technology Institute, Korea Atomic Energy Research Institute, Jeongeup 56212, Republic of Korea

[3] Department of Biosystem and Bioengineering, KRIBB School of Biotechnology, University of Science and Technology (UST), Daejeon 34113, Republic of Korea

[4] Present address: National Key Laboratory of Plant Molecular Genetics, CAS Center for Excellence in Molecular Plant Sciences, Chinese Academy of Sciences, Shanghai 200032, China

Correspondence: Jiyoung Lee, jiyoung1@kribb.re.kr

Running Head: ***Jeongeuplla avenae*** **gen. nov., sp. nov.**

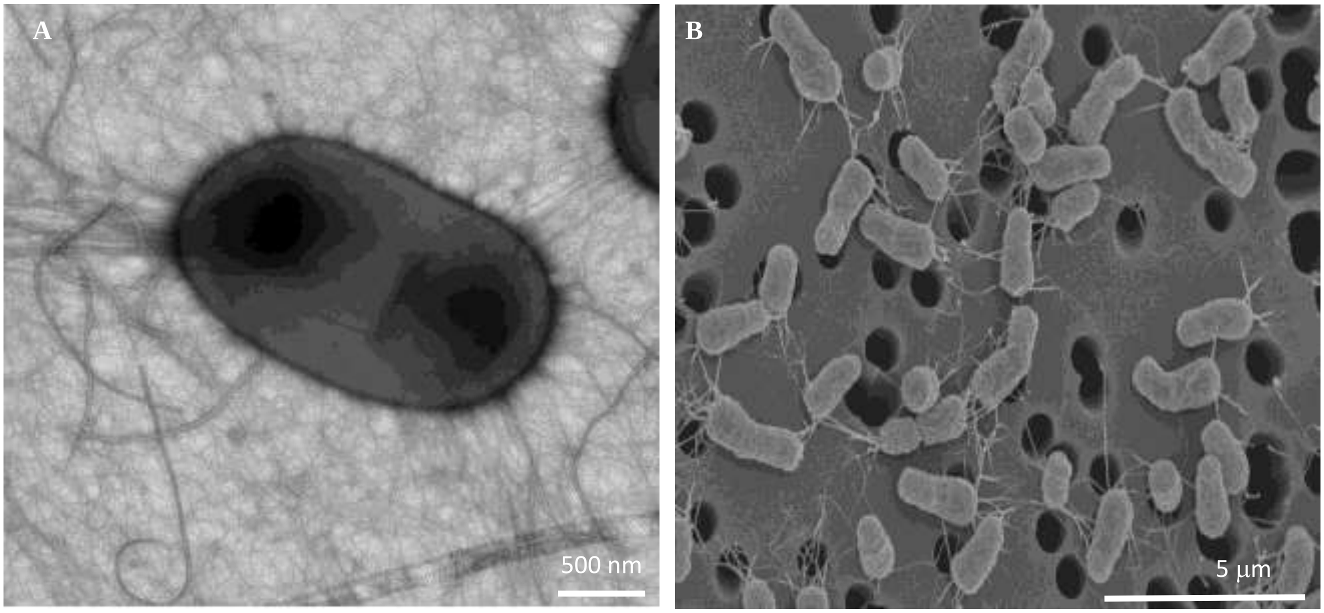

Fig S1. Transmission electron microscope (TEM) and scanning electron microscope (SEM) image of strain DY-R2A-6^{T}. Scale bars, 500 nm and 5 μm.

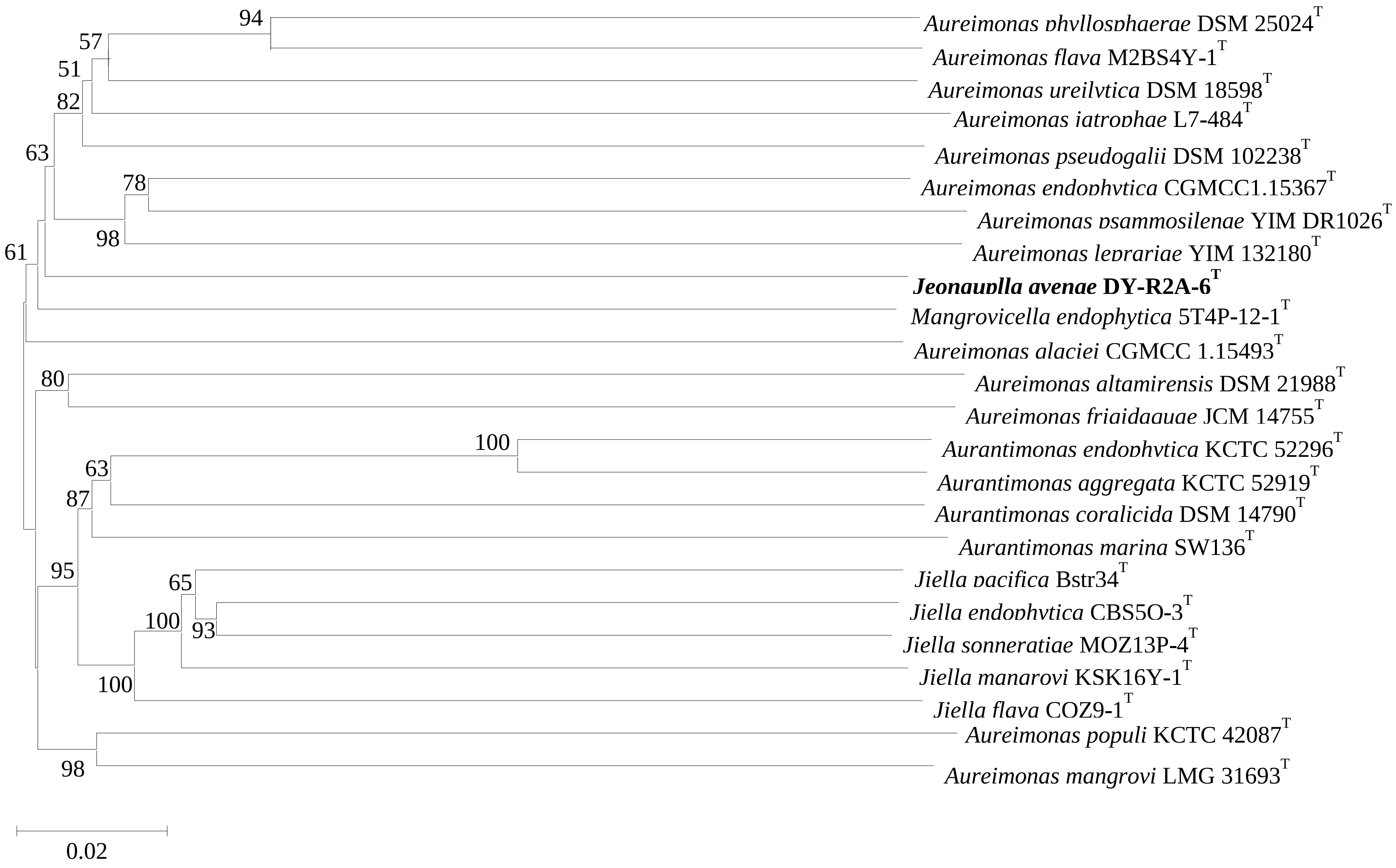

Fig S2. Tree inferred with FastME 2.1.6.1 [1] from GBDP distances calculated from genome sequences. The branch lengths in the tree are scaled in terms of GBDP distance formula d5. The values displayed above the branches are GBDP pseudo-bootstrap support values, with a threshold of 50% from 100 replications, and an average branch support of 92.7%. The midpoint was chosen as the root of the tree [2].

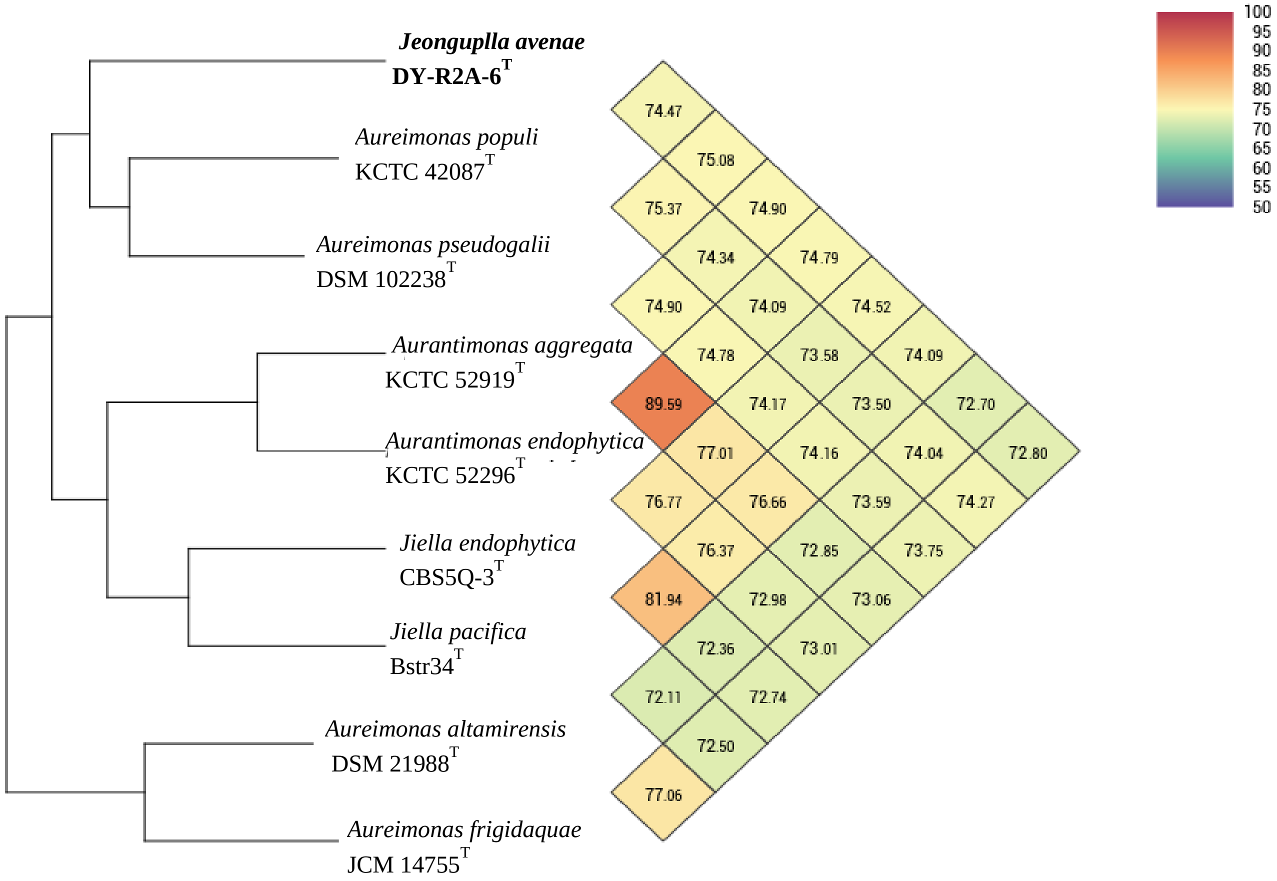

Fig S3. Visualizing a Heatmap of OrthoANI values calculated with OAT software to compare DY-R2A-6^T and closely related strains. The color code used in the visualization represents the closest species as green and the farthest as red. On the left side, an unweighted pair group method with an arithmetic mean (UPGMA) dendrogram based on OrthoANI values of eight species is presented.

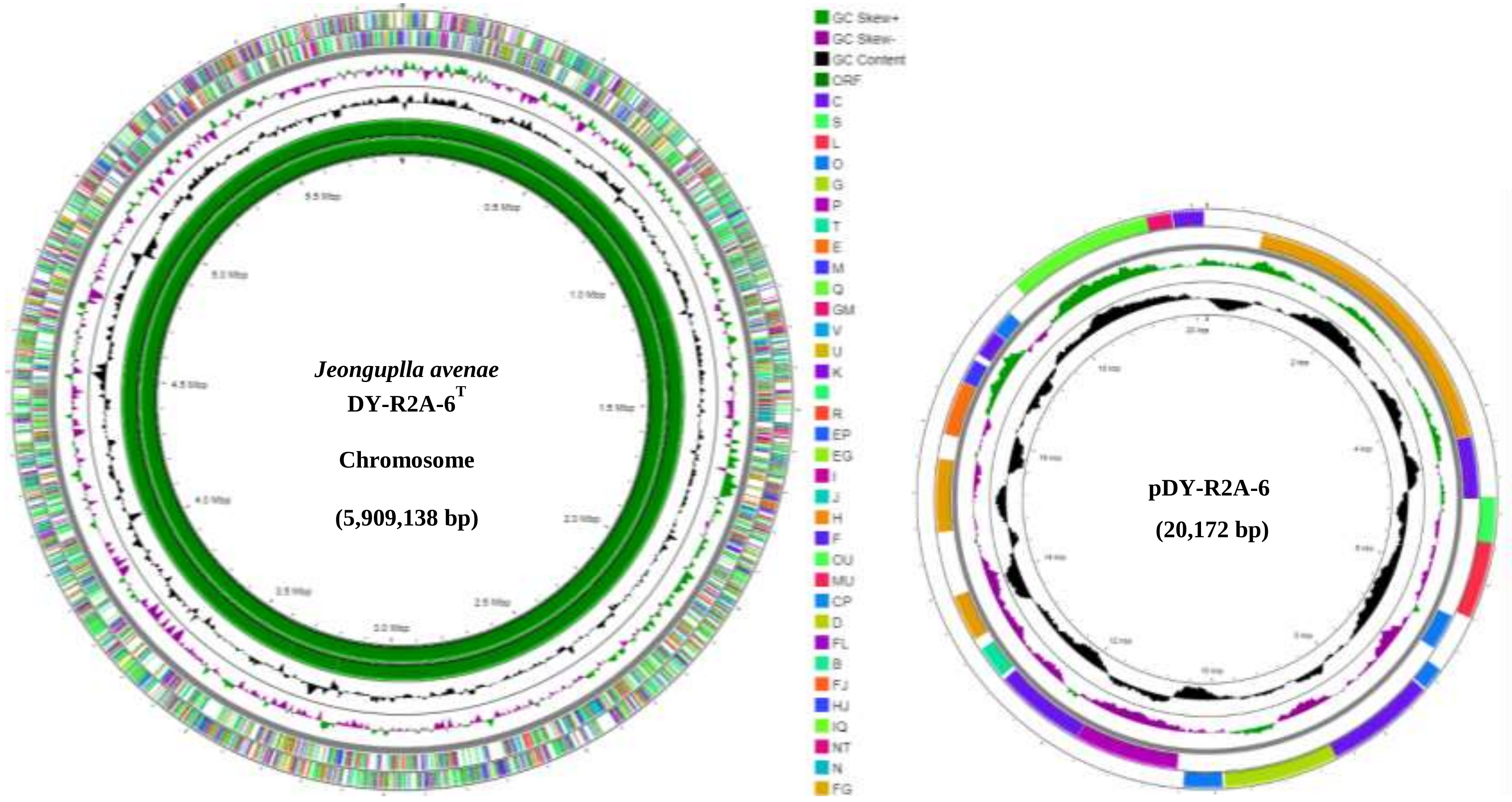

Fig S4. Map of the DY-R2A-6^T genome (left) and plasmid (right) generated with CGView. Marked characteristics are shown from the outside to the center. Rings 1 and 2 show cluster orthologous group (COG) annotation in the forward and reverse directions, respectively. Ring 3 shows the GC skew, while ring 4 shows the G+C % content plot. Rings 5 and 6 show the NCBI annotation in the forward and reverse

directions. The COG categories are: A, RNA processing and modification; B, chromatin structure and dynamics; C, energy production and conversion; D, cell cycle control, cell division, and chromosome partitioning; E, amino acid transport and metabolism; F, nucleotide transport and metabolism; G, carbohydrate transport and metabolism; H, coenzyme transport and metabolism; I, lipid transport and metabolism; J, translation, ribosomal structure, and biogenesis; K, transcription; L, replication, recombination, and repair; M, cell wall/membrane/envelope biogenesis; N, cell motility; O, post-translational modification, protein turnover, chaperones; P, inorganic ion transport and metabolism; Q, secondary metabolite biosynthesis, transport, and catabolism; R, general function prediction only; S, function unknown; T, signal transduction mechanisms; U, intracellular trafficking, secretion, and vesicular transport; V, defense mechanisms; W, extracellular structures; X, mobilome: prophages, transposons; and Z, cytoskeleton.

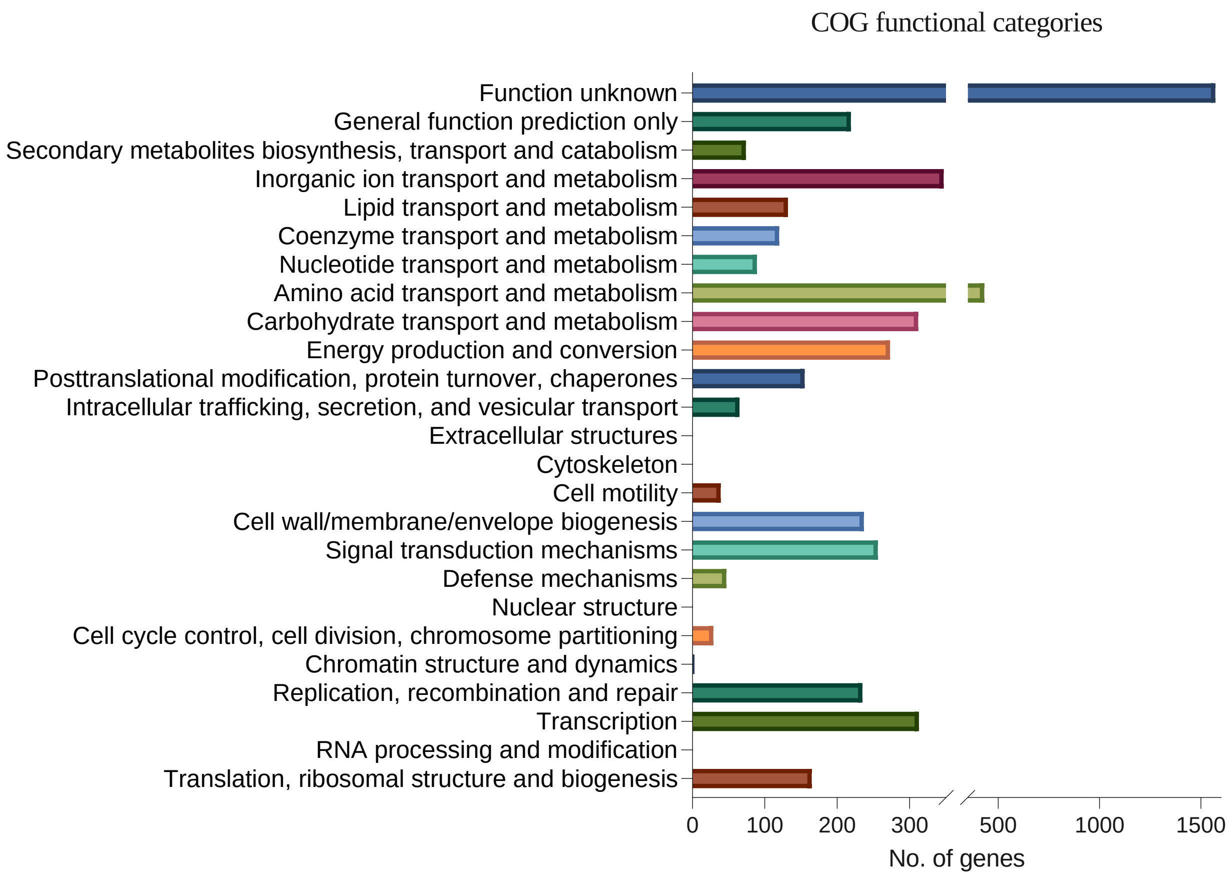

Fig S5. The cluster of orthologous groups (COG) classification of putative proteins in the whole-genome sequence of strain DY-R2A-6^{T}.

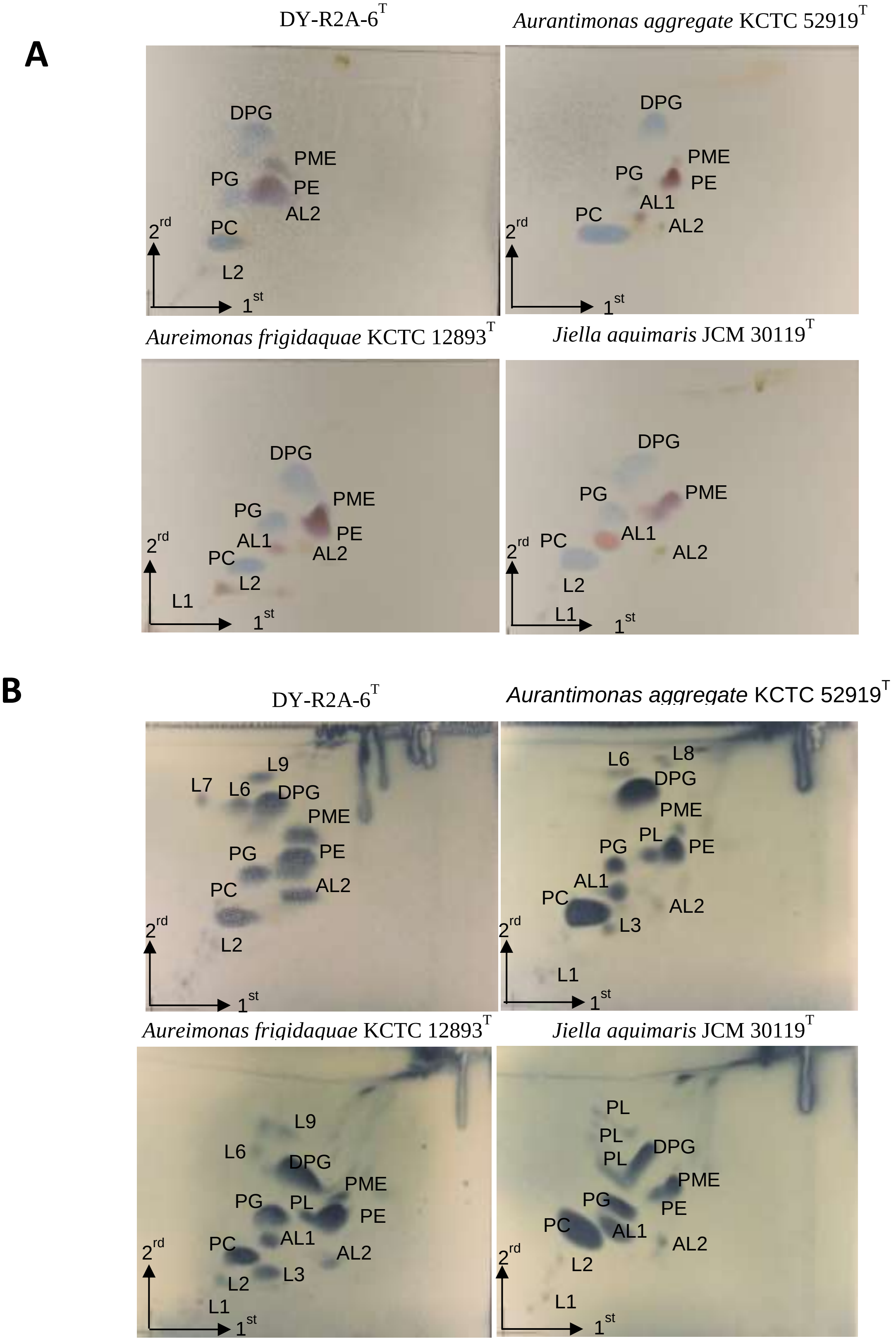
A
DY-R2A-6T
Aurantimonas aggregate KCTC 52919T
DPG
PME
PG
PE
AL2
PC
L2
AL1
2rd
1st
Aureimonas frigidaquae KCTC 12893T
Jiella aquimaris JCM 30119T
L1
B
L9
L7
L6
L8
L3
PL

Fig S6. Two-dimensional TLC of total polar lipids of strain DY-R2A-6^T and closely related type strains. (A) Phospholipids and aminolipids were detected by spraying with molybdenum blue and ninhydrin reagents, respectively. (**B)** Total lipids were detected by spraying the plate with phosphomolybdic acid reagent. DPG**,** diphosphatidylglycerol; PE, phosphatidylethanolamine; PME, phosphatidylmonomethylethanolamine; PG, phosphatidylglycerol; PC, phosphatidylcholine; AL1-2, unknown aminolipids; L1-L9, unknown polar lipids.

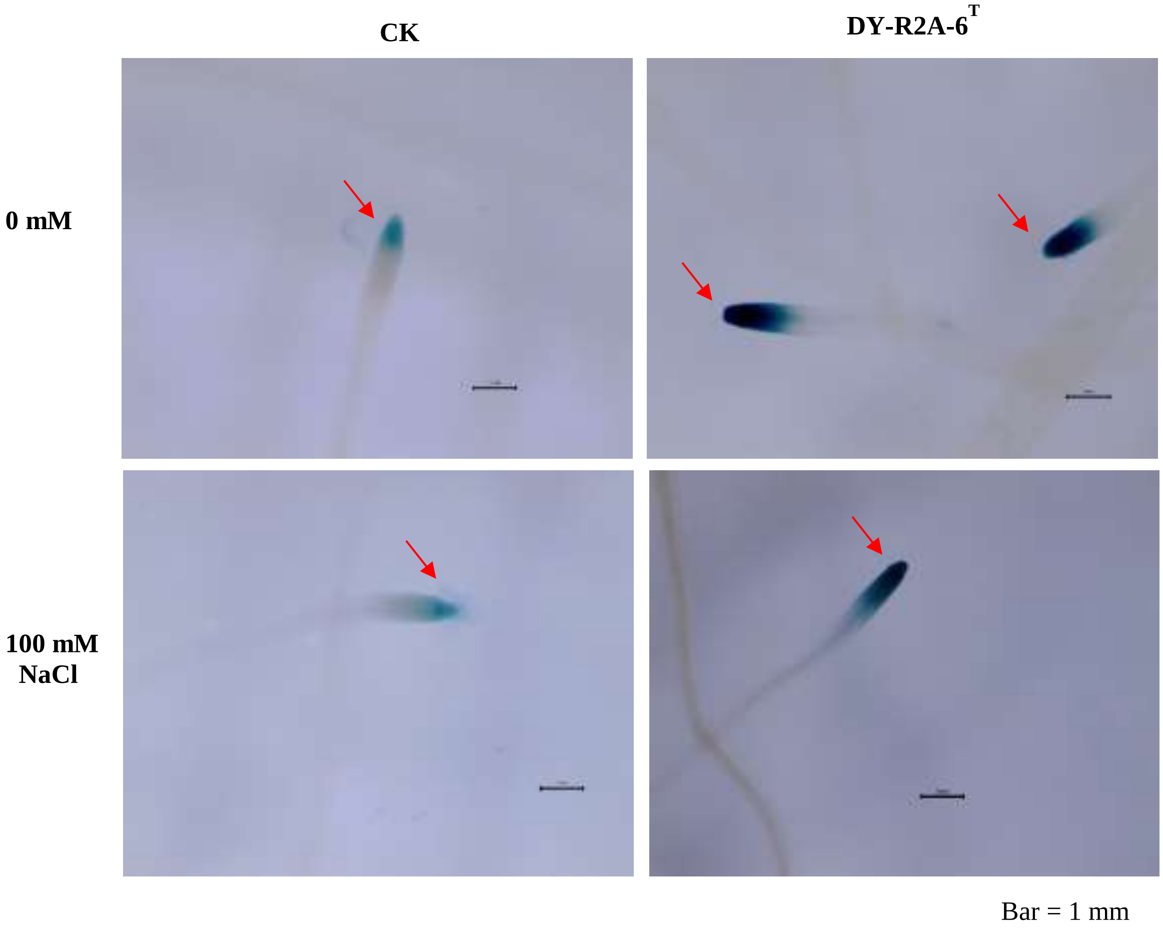

Fig S7. GUS activity in DR5:GUS Arabidopsis lines in salt stress condition. Arabidopsis seedlings were transferred onto 1/2MS medium supplemented with 0 and 100 mM NaCl, and co-cultivated with or without DY-R2A-6^T for additional 10 days cultivation seedlings were subjected to β-glucuronidase (GUS) staining. A representative picture of each treatment is shown root tips. Scale bar, 1 mm.

Table S1. The genome features of all type species in family *Aurantimonadaceae* used in this study.

Type species	N_{50}	Completeness (%)	Contamination (%)	Quality	NCBI assembly number	Genome length (Mbp)	DNA G+C content (%)	Number of contigs
Jeonguplla avenae DY-R2A-6^{T}	5,909,198	100.0%	1.9%	90.5%	GCA_026723785.1	5,909,198	69.1%	1
Aurantimonas manganoxydans SI85-9A1^{T}	4,285,343	99.1%	0.9%	95.5%	GCA_000153465.1	4,326,814	66.7%	22
Aureimonas ureilytica DSM 18598^{T}	452,612	100.0%	3.8%	81.0%	GCA_000382705.1	5,087,183	67.3%	44
Aurantimonas coralicida DSM 14790^{T}	181,777	100.0%	0.9%	95.5%	GCA_001463825.1	4,603,216	66.7%	93
Aureimonas altamirensis DSM 21988^{T}	305,097	99.1%	0.9%	94.6%	GCA_001463885.1	4,187,548	64.8%	32
Aureimonas frigidaquae JCM 14755^{T}	256,739	98.1%	0.9%	93.6%	GCA_001463905.1	4,098,233	66.1%	30
Aureimonas ureilytica DSM 18598 ^{T}	157,113	100.0%	3.8%	81.0%	GCA_001463945.1	5,097,296	67.3%	106
Aureimonas flava M2BS4Y-1^{T}	424,277	99.1%	0.9%	94.6%	GCA_003583935.1	4,090,174	70.0%	26
Jiella endophytica CBS5Q-3^{T}	669,366	94.6%	21.6%	low	GCA_004519335.1	5,252,418	66.5%	28
Aureimonas fodinaquatilis CAU 1482^{T}	1,264,131	99.1%	0.9%	94.6%	GCA_008369445.1	3,936,980	56.7%	8
Aureimonas leprariae YIM 132180^{T}	213,101	99.1%	0.9%	94.6%	GCA_008802405.1	4,779,519	68.4%	40
Aureimonas psammosilenae YIM DR1026^{T}	347,487	99.1%	0.9%	94.6%	GCA_009176975.1	4,892,941	64.6%	50
Jiella pacifica 40Bstr34^{T}	187,795	99.1%	0.9%	94.6%	GCA_010500815.1	5,774,732	65.7%	74
Aurantimonas aggregate KCTC 52919^{T}	186,498	99.1%	0.9%	94.6%	GCA_010500835.1	4,861,770	66.6%	95
Aureimonas mangrove LMG 31693^{T}	3,485,124	100.0%	1.9%	90.5%	GCA_014058705.1	3,485,124	66.8%	1
Aureimonas jatrophae L7-484^{T}	618,531	98.1%	1.9%	88.6%	GCA_014196395.1	4,596,631	67.8%	20
Aureimonas phyllosphaerae DSM 25024^{T}	475,420	100.0%	1.9%	90.5%	GCA_014196405.1	4,702,309	67.5%	46
Aureimonas pseudogalii DSM 102238^{T}	255,520	100.0%	1.9%	90.5%	GCA_014196835.1	5,002,150	67.9%	53
Aureimonas endophytica CGMCC 1.15367 ^{T}	692,434	100.0%	0.9%	95.5%	GCA_014642635.1	5,332,506	68.3%	45
Aureimonas glaciei CGMCC 1.15493^{T}	318,995	99.1%	1.9%	89.6%	GCA_014643375.1	5,642,654	66.8%	33
Aurantimonas marina SW1361^{T}	191,515	100.0%	0.9%	95.5%	GCA_017183135.1	4,028,999	64.3%	66
Jiella flava CQZ9-1^{T}	254,147	100.0%	0.9%	95.5%	GCA_017349315.1	4,162,933	63.8%	49
Jiella sonneratiae MQZ13P-4^{T}	199,219	100.0%	0.0%	100.0%	GCA_017353515.1	5,153,243	68.1%	76
Aureimonas populi KCTC 42087^{T}	3,822,311	99.1%	0.9%	94.6%	GCA_017815515.1	3,822,311	68.2%	1

Aureimonas altamirensis C2P003^T	4,592,981	99.1%	0.9%	94.6%	GCA_021228915.1	4,592,981	66.3%	1
Aurantimonas endophytica KCTC 52296^T	692,717	99.1%	0.9%	94.6%	GCA_024105745.1	5,043,873	66.6%	12
Aureimonas jatrophae L7-484^T	397,844	98.1%	1.9%	88.6%	GCA_900104035.1	4,590,703	67.8%	25
Aureimonas phyllosphaerae L9-753^T	203,496	100.0%	1.9%	90.5%	GCA_900113065.1	4,715,690	67.5%	62
Aureimonas altamirensis DSM 21988^T	1,054,710	99.1%	0.9%	94.6%	GCA_900141975.1	4,190,965	64.8%	11

Table S2. The AAI values among the strain DY-R2A-6^T and the closely related strains in the family *Aurantimonadaceae*. Data were calculated from the AAI calculator of Kostas Lab.

1, *Jeonguplla avenae* DY-R2A-6^T; 2, *Aurantimonas aggregate* KCTC 52919^T; 3, *Aureimonas frigidaquae* JCM 14755^T; 4, *Jiella pacifica* 40Bstr34^T; 5, *Aureimonas pseudogalii* DSM 102238^T; 6, *Aureimonas altamirensis* DSM 21988^T; 7, *Jiella endophytica* CBS5Q-3^T; 8, *Aureimonas endophytica* CGMCC 1.15367^T; 9, *Aureimonas populi* KCTC 42087^T.

	1	2	3	4	5	6	7	8	9
1	-								
2	64.2%								
3	61.8%	62.4%							
4	61.2%	67.6%	61.0%						
5	63.7%	63.4%	64.8%	61.8%					
6	61.8%	62.8%	75.6%	60.9%	64.7%				
7	62.1%	67.8%	61.1%	79.2%	62.0%	61.2%			
8	63.8%	90.8%	62.2%	67.0%	63.6%	63.3%	68.0%		
9	63.4%	64.5%	65.8%	62.4%	65.9%	66.4%	62.3%	64.3%	

Table S3. The ANI (%, lower triangle in bold) and dDDH (%, upper triangle) values among the strain DY-R2A-6^T and the closely related strains in the family *Aurantimonadaceae*. Data were calculated from the ANI calculator by Kostas Lab and GGDC web server. 1, *Jeonguplla avenae* DY-R2A-6^T; 2, *Aurantimonas aggregate* KCTC 52919^T; 3, *Aureimonas frigidaquae* JCM 14755^T; 4, *Jiella pacifica* 40Bstr34^T; 5, *Aureimonas pseudogalii* DSM 102238^T; 6, *Aureimonas altamirensis* DSM 21988^T; 7, *Jiella endophytica* CBS5Q-3^T; 8, *Aureimonas endophytica* CGMCC 1.15367^T; 9, *Aureimonas populi* KCTC 42087^T.

	1	2	3	4	5	6	7	8	9
1		20.2%	19.7%	20.5%	20.4%	20.3%	20.5%	20.4%	20.0%
2	78.7%		19.9%	21.5%	19.9%	19.9%	21.4%	38.0%	20.0%
3	78.0%	77.9%		20.4%	19.7%	20.2%	20.1%	20.0%	19.7%
4	78.4%	79.7%	77.5%		20.2%	20.6%	24.8%	21.5%	20.2%
5	79.2%	78.4%	78.1%	78.1%		19.9%	20.2%	20.0%	20.1%
6	78.1%	77.8%	79.9%	77.7%	78.1%		20.9%	19.6%	19.9%
7	78.6%	79.7%	77.6%	82.7%	78.3%	77.8%		21.4%	20.1%
8	78.7%	88.9%	77.8%	79.6%	78.5%	77.9%	79.6%		19.9%
9	78.5%	78.4%	78.5%	78.3%	79.1%	78.5%	78.0%	78.4%	

Table S4. Presence of secondary metabolite biosynthetic gene clusters in the genome sequence of strain DY-R2A-6^T, as detected using antiSMASH.

Type	From	To	smBGC type	Most similar known cluster	Similarity	Core biosynthetic gene	Additional biosynthetic gene
T1PKS	277,637	318,698	Bacillomycin D	Polyketide+NRP:Lipopeptide	20%	1	6
Terpene	713,490	736,308	Carotenoid	Terpene	83%	2	8
NAGGN	1,875,260	1,890,019	-	-	-	3	4
4T1PKS,NRPS-like,terpene	2,702,821	2,771,623	-	-	-	3	10
Hserlactone	3,802,191	3,822,883	-	-	-	1	2
RiPP-like	4,547,822	4,557,314	-	-	-	1	1
Redox-cofactor	5,004,076	5,026,236	-	-	-	3	3
Thioamitides	5,628,740	5,650,724	CittilinA/cittilin B	RiPP	7%	2	1

1. Lefort, V, Desper, R, Gascuel, O, 2015. FastME 2.0: A Comprehensive, Accurate, and Fast Distance-Based Phylogeny Inference Program. Mol Biol Evol 32:2798-800. doi: 10.1093/molbev/msv150
2. Bhattacharjee, A, Bayzid, MS, 2020. Machine learning based imputation techniques for estimating phylogenetic trees from incomplete distance matrices. BMC Genomics 21:497. doi: 10.1186/s12864-020-06892-5
